# An ensemble of parameters from a robust Markov-based model reproduces L-type calcium currents from different human cardiac myocytes

**DOI:** 10.1371/journal.pone.0266233

**Published:** 2022-04-05

**Authors:** Gustavo Montes Novaes, Enrique Alvarez-Lacalle, Sergio Alonso Muñoz, Rodrigo Weber dos Santos

**Affiliations:** 1 Graduate Program in Computational Modeling, Federal University of Juiz de Fora, Juiz de Fora, MG, Brazil; 2 Department of Physics, Universitat Politècnica de Catalunya-BarcelonaTech, Barcelona, Spain; 3 Department of Computation and Mechanics, Federal Center of Technological Education of Minas Gerais, Leopoldina, MG, Brazil; University at Buffalo - The State University of New York, UNITED STATES

## Abstract

The development of modeling structures at the channel level that can integrate subcellular and cell models and properly reproduce different experimental data is of utmost importance in cardiac electrophysiology. In contrast to gate-based models, Markov Chain models are well suited to promote the integration of the subcellular level of the cardiomyocyte to the whole cell. In this paper, we develop Markov Chain models for the L-type Calcium current that can reproduce the electrophysiology of two established human models for the ventricular and Purkinje cells. In addition, instead of presenting a single set of parameters, we present a collection of set of parameters employing Differential Evolution algorithms that can properly reproduce very different protocol data. We show the importance of using an ensemble of a set of parameter values to obtain proper results when considering a second protocol that suppresses calcium inactivation and mimics a pathological condition. We discuss how model discrepancy, data availability, and parameter identifiability can influence the choice of the size of the collection. In summary, we have modified two cardiac models by proposing new Markov Chain models for the L-type Calcium. We keep the original whole-cell dynamics by reproducing the same characteristic action potential and calcium dynamics, whereas the Markov chain-based description of the L-type Calcium channels allows novel small spatial scale simulations of subcellular processes. Finally, the use of collections of parameters was crucial for addressing model discrepancy, identifiability issues, and avoiding fitting parameters overly precisely, i.e., overfitting.

## Introduction

The large variability in action potential (AP) and contraction responses in cardiomyocytes not only across different species and cells [1] but also across different individuals [2], has given rise to a significant effort to develop models that can properly mimic different action potential dynamics [1, 3–5].

These differences in the cell behavior can be linked with pathologies associated with Atrial Fibrillation [6], Brugada Syndrome [7], or Long-QT [8], among others. The search for these general pro-arrhythmic characteristics in computational models is directly related to the development of proper parameter estimations in order to develop a better understanding of specific channelopathies related to genetic mutations [9]. Experimental measures of the average effect of particular gene mutations such as ΔKPQ or KCNQ1 [10] or genetically modified organism [11] to study dysfunction in contraction due to problems in calcium handling can be used to find key modifications in the parameters space or the need for changes in the structural properties of channels, pumps, or receptors. Therefore, modeling structures at the channel level that can be integrated into different models and properly reproducing different experimental data is of great importance.

Modeling cardiomyocyte channels as Markov Chains presents a clear advantage compared with Hodgkin-Huxley (HH) type models, as the former allows the possibility to model intracellular dynamics. The integration of the variability to analyze cell data from subcellular calcium imaging, patch-clamp, and small tissue leads to computational cellular models that span multiple scales. Specifically, subcellular data of spark activity and structure, or spatial differences in the calcium transient behavior depending on the level of t-tubule structure, cannot be properly analyzed with a HH framework making it impossible to scale up from the micrometer to the millimeter scale. This is especially relevant in the study of excitation-contraction coupling via calcium handling. The key element of this handling is the calcium release units, where a small number of L-type Calcium Channels (LCC) face a cluster of Ryanodine Receptors [12]. A Markov Chain model structure can be used both in a subcellular model of the cardiomyocyte [13] and in a whole-cell approach where these units are coarse-grained. Similarly, the variability in parameters space needed to deal with the variability in behavior is efficiently dealt with by Markov Chain models. More importantly, the parameter space of the different populations can be directly linked with the necessary stochastic nature of calcium handling at the micrometer scale. Following this approach, the analysis of these stochastic subcellular properties have been recently employed to study large-scale organ behavior by introducing the probability distribution of subcellular models into whole-cell models [14].

The inclusion of channel variability into whole-cell organs is particularly relevant to study the effect of differences in calcium handling across animals and patients. Doing so necessarily requires introducing Markov Chain models that can properly reproduce the stochastic subcellular behavior. Different properties of the LCC, the Ryanodine Receptors, the Sodium-Calcium exchanger (NCX), and the SERCA pump have been related with calcium alternans [15, 16]. Similarly, the relevance of calcium handling on the development of Atrial Fibrillation (AF) and its direct association with Heart Failure is very well established [17, 18].

The relevance of the LCCs and the Ryanodine Receptors in calcium handling is very well-known. Calcium-Induced Calcium-Release in the calcium release unit scale is the key to understand calcium alternans [19] in ventricles. Not as a period double-bifurcation, as it is often stated in whole-cell models, but as an order-disorder Ising-like transition [19]. The Ryanodine Receptors are normally portraited as a Markov Chain model with two [20], four [21], or more states in whole-cell models. The same structure is used for subcellular and whole-cell models allowing for direct comparison between them. A whole-cell model where the Ryanodine Receptor has multiple states and its transitions are not stochastic but deterministic can be understood as the limit of having the different calcium release units tightly coupled by calcium diffusion. However, this is not the case for the LCC in most whole-cell models. This prevents the easy comparison among different length scales and the generation of a collection of parameters that can deal with the different sources of experimental data.

There is a great interest in producing L-type Calcium models that can be integrated into subcellular, whole-cell, and tissue models. The purpose of this manuscript is the development of Markov Chain models that can reproduce AP and intracellular calcium signals both from experiments or from already established models of particular animals or cells. In addition, instead of presenting a single set of parameters for the new model, we propose the use of a collection of parameters, labeled as population, to overcome the limitations usually associated with overfitting.

The goal is to find a collection of the parameters of the L-type Calcium current (I_CaL_) model that can properly match very different protocol data. We show that proper penalty functions and evolutionary algorithms can generate a collection of parameters that can reproduce different protocols. We explain that the approach is sound by replacing two different HH Gate-based I_CaL_ models of the ventricle and Purkinje human cells with a Markov Chain-based (MC-based) model. We first reproduce all the original model outputs in each case, showing that the Markov Chain structure and the algorithm can match any particular data source. More importantly, we implement different experimental-like protocols to the gate-based models and show how considering a collection of sets of parameter approaches gives the system enough flexibility to fit different data. One of the protocols simulates the condition of Calmodulin mutations associated with long QT syndrome, which is known to promote proarrhythmic behavior in ventricular myocytes.

We develop a general procedure to generate a collection of parameters. We use well-establish models to make the reasoning clear. However, the same method can be used to reproduce different experimental data on calcium handling, not only by measuring the interaction between calcium and voltage measures but also from single-channel data or any other type of interaction or protocol employed.

In addition, we also discuss how model discrepancy, data availability, and parameter identifiability can influence the choice of the size of the collection of parameters. By acknowledging the existence of model discrepancy and identifiability issues, the use of higher tolerances after the fitting process may avoid fitting parameters overly precisely, i.e., overfitting.

## Materials and methods

### Cardiac models

An extensively employed mathematical model based in a Markov Chain (MC) description to simulate the electrophysiology of cardiac cells is described in Mahajan et al. [22]. The main objective of the model presented in Mahajan et al. [22] is the accurately reproduction of the cardiac AP and the Intracellular Calcium Cycling at rapid heart rates. Starting from a previous rabbit cardiac model [21], Mahajan et al. [22] modified the formulation of the I_CaL_ current by replacing it with a seven-state Markovian model. The I_CaL_ equation proposed by Mahajan et al. [22] reads

$$\begin{array}{c} I_{CaL}=P_{o}\times {\bar{g}}_{Ca}\frac{4P_{Ca}VF^{2}}{RT}\frac{c_{s}e^{2(VF/RT)}-0.341{\left[Ca^{2+}\right]}_{o}}{e^{2(VF/RT)}-1}, \end{array}$$ (1)

where *P*_*o*_ is the Opening fraction of the channels (represented by the Markovian Open state), ${\bar{g}}_{Ca}$ is the maximum conductivity parameter, *V* is the transmembrane potential, and *c*_*s*_ is the submembrane calcium concentration. The other terms are parameters of the model or physical constants.

However, there are also many models that use HH Gate-based descriptions for the I_CaL_ formulations. Such models have been extensively employed in the modeling of cardiac tissue at large spatial scales. We consider here two typical examples of these models: [23, 24] employed for ventricular tissue and Purkinje Fibers, respectively.

The first consolidated cardiac computational model is the Ten Tusscher and Panfilov [23]. This model, initially developed in [25], simulates the electrophysiology of the left ventricular cells of humans. Different from the description adopted by Mahajan et al. [22], the Ten Tusscher and Panfilov [23] model uses HH Gate-based structures to compose the I_CaL_ formulation. It is based in the combination of three voltage-dependent gates with another calcium-dependent gate to simulate the opening fraction of the LCC.

The second consolidated cardiac model used here is the Stewart et al. [24]. The model is based on Ten Tusscher and Panfilov [23] with modifications to simulate the electrophysiology of the human Purkinje fibers cells.

Both models, Ten Tusscher and Panfilov [23], and Stewart et al. [24], adopted the HH Gate-based approach to simulate the opening fraction dynamics of the I_CaL_ current. Rearranging the terms, I_CaL_ equation for both models reads

$$\begin{array}{c} I_{CaL}=dff_{2}f_{cass}\times G_{CaL}\frac{4\left(V-15\right)F^{2}}{RT}\frac{0.25c_{ss}e^{2(V-15)F/RT}-{\left[Ca^{2+}\right]}_{o}}{e^{2(V-15)F/RT}-1}, \end{array}$$ (2)

where *d*, *f* and *f*_2_ are the three voltage-dependent gates, and *f*_*cass*_ is the calcium-dependent one; *G*_*CaL*_ is the maximum conductance of the current; *V* is the transmembrane potential; and *c*_*ss*_ is the diadic subspace calcium concentration. The other terms are model parameters or physical constants.

**Fig 1:**
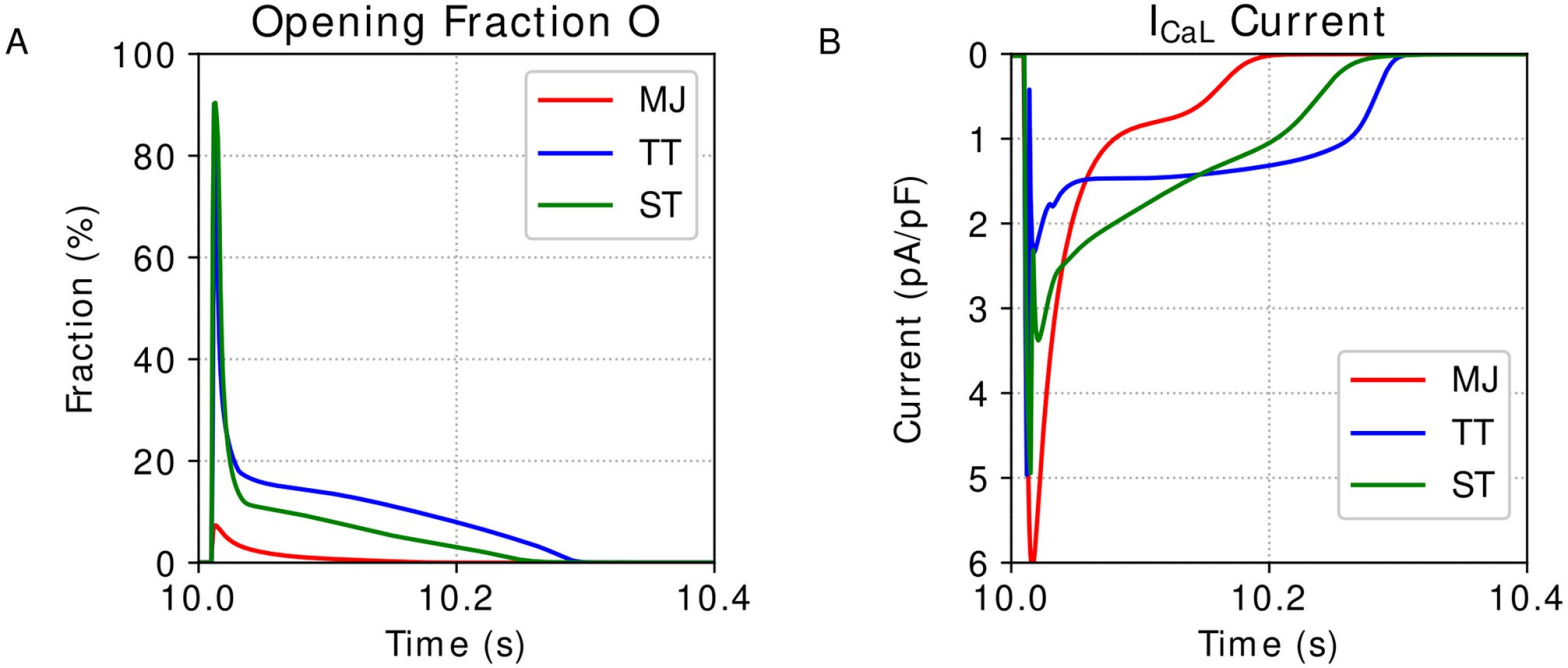
Cardiac models outputs. Output dynamics generated by the simulation of the models Mahajan et al. [22] (MJ), Ten Tusscher and Panfilov [23] (TP), and Stewart et al. [24] (ST). A: Opening Fraction, *O*. B: I_CaL_ current.

As can be seen, the three cited models, Mahajan et al. [22], Ten Tusscher and Panfilov [23], and Stewart et al. [24], simulate the same phenomenon, and, disregarding the parameters and the values of the physical constants, they use the same equation to simulate the I_CaL_ current. Furthermore, it is possible to read the I_CaL_ equation of the three models as a multiplication of two terms: *I*_*CaL*_ = *O* × *I*_*max*_; where *O* is the channels Opening fraction and the *I*_*max*_ is the maximum current value when all channels are open. In Mahajan et al. [22] model, this opening fraction term is represented by the Markovian state P_o_ and reaches peak levels around 10% of opening. On the other hand, in both Ten Tusscher and Panfilov [23], and Stewart et al. [24] models, this opening fraction is represented by the multiplication of the four gates *d*, *f*, *f*_2_, *f*_*cass*_, and reach the peak of around 90% in the opening levels. In the first moment, this difference in the amplitude of the opening fraction values can look weird. However, it is important to highlight the different natures that each model assumes. For instance, Mahajan et al. [22] focus on models for cardiac cells of rabbits, whereas Ten Tusscher and Panfilov [23], and Stewart et al. [24] propose models for cardiac cells of humans. Therefore, the difference observed in Fig 1, showing the dynamics of the opening fraction, *O*, and the L-type Calcium current, I_CaL_, over the time for the models from Mahajan et al. [22], Ten Tusscher and Panfilov [23], and Stewart et al. [24], may be due to the differences between rabbits and humans.

As stated above, we consider two different approaches to model the opening fraction dynamics. Majahan et al. [22] uses a MC-based structure, while Ten Tusscher and Panfilov [23], and Stewart et al. [24] use a Gate-based formulation. However, as discussed by Mahajan et al. [22], the use of an MC-based approach naturally models the ion channel biophysical properties in terms of molecular transitions between discrete conformation states.

The first goal of this work is to propose and test a new MC-based I_CaL_ model that can replace the opening fraction, *O*, composed by the four gates *d*, *f*, *f*_2_, *f*_*cass*_, originally used in Ten Tusscher and Panfilov [23] (TP), and Stewart et al. [24] (ST) models.

### Markov chain-based model

Considering that a single HH gate *g* can assume only two states (O—Open, and C—Close), we can generate a Markovian model for this gate, calculating the transition rates *g*_+_ (rate of the transition *C* → *O*) and *g*_−_ (rate of the transition *O* → *C*) as

$$\begin{array}{c} g_{+}=\frac{g_{\infty }}{\tau_{g}} \end{array}$$ (3)

and

$$\begin{array}{c} g_{-}=\frac{1-g_{\infty }}{\tau_{g}}, \end{array}$$ (4)

where *g*_∞_ and *τ*_*g*_ are equations defined by the HH Gate-based formalism [26].

**Fig 2:**
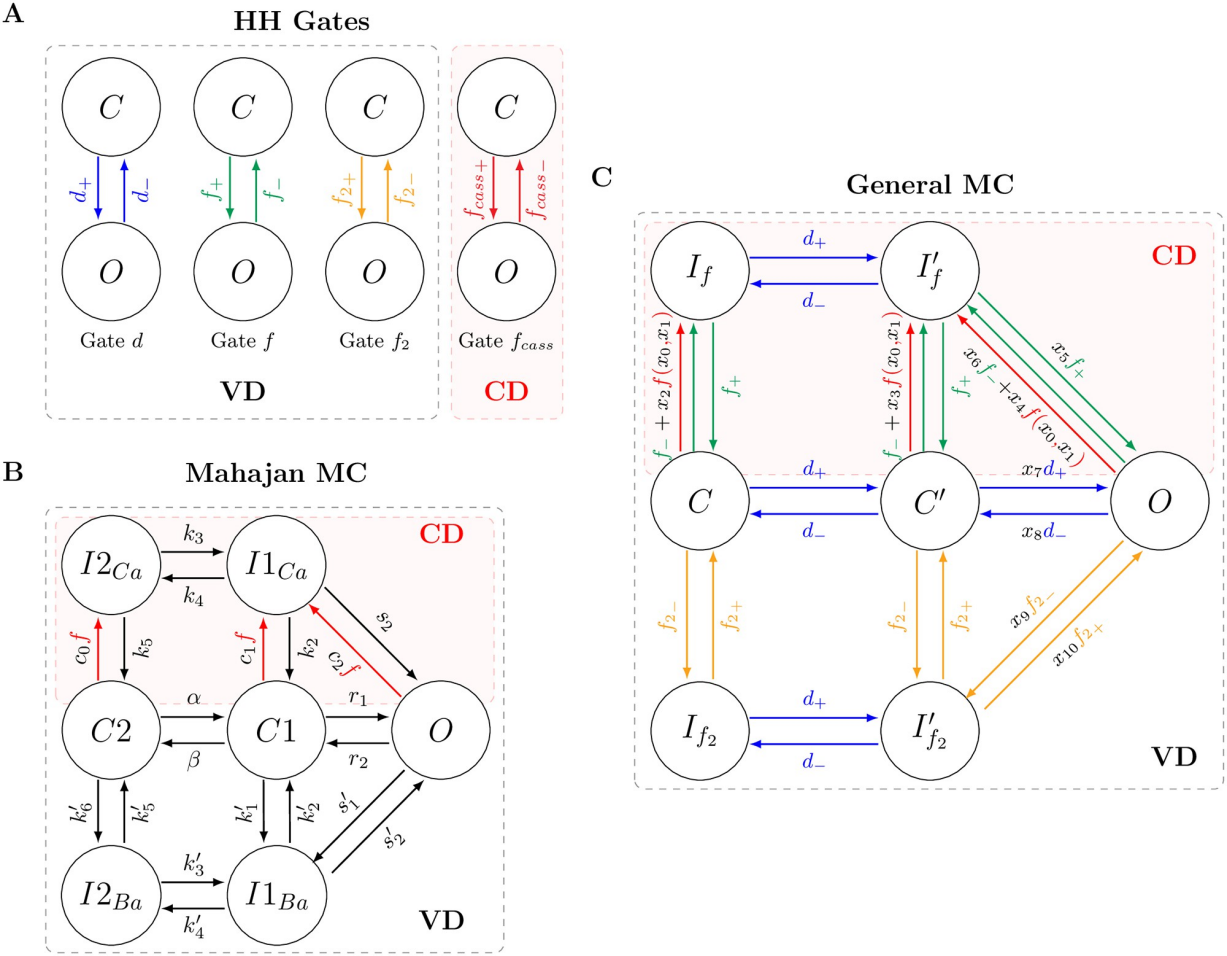
Schematic representations of the three markov chain structures considered in this study. A: The four independent Markov Chains for each gate of the models Ten Tusscher and Panfilov [23] or Stewart et al. [24] considering only two possible states, Open (*O*) and Close (*C*) for each one. B: The original structure of the Markov Chain used by Mahajan et al. [22] to simulate the I_CaL_ phenomenon. C: The proposed Markov Chain as a combination of the Hodgkin-Huxley formalism rates and the Mahajan et al. [22] topology, to replace the gates in the Ten Tusscher and Panfilov [23] and Stewart et al. [24] models. The MC transitions generated considering the HH gates *d*, *f*, and *f*_2_ are shown respectively in blue, green, and yellow. The calcium-dependent function *f* and the rates associated with the calcium concentration are shown in red. The set of parameters ***x*** used to fit both calcium-dependent rates (parameters *x*_0_ to *x*_4_) and voltage-dependence rates (parameters *x*_5_ to *x*_10_) are shown in black.

Applying this analysis to the four gates of both TP, and ST models, it is possible to calculate all the rates that control the dynamics between the Open and Close states for each gate. Fig 2A illustrates these single MCs for each of the four gates and their respective transition rates. To propose a MC-based model for the cardiac models TP, and ST, we use these eight transition rates.

Once we defined the transition rates, we need to set the MC topology. Here, we adopt the minimal seven-state MC arrangement proposed by Mahajan et al. [22]. Fig 2B illustrates the original Mahajan et al. [22] MC for the I_CaL_ formulation.

Thus, to transform the original gates from the Gate-based models into the MC-based formulation, we combine the rates of the HH formalism, Fig 2A, with the consolidated I_CaL_ MC-based topology, Fig 2B. The result of this combination is the new proposed MC-based model presented in Fig 2C. In addition, to adjust this new MC-based model to the original human models, we introduce a set of eleven parameters that multiply some of the MC transition rates: $x=\{x_{i}|x_{i}\in \mathbb{R}\;\text{and}\;i=0-10\}$.

The first five fitting parameters, *x*_0_ up to *x*_4_, are used to handle the MC calcium sensitivity. Considering the MC top layer, states *I*_*f*_ and $I_{f}^{\prime }$, as the calcium-dependence layer, these five parameters are related to calcium-dependence. The first two parameters, *x*_0_ and *x*_1_, compose the calcium-dependent equation

$$\begin{array}{c} f\left(c,x_{0},x_{1}\right)=\frac{1}{1+{\left(x_{0}{\bar{c}}_{p}/c\right)}^{x_{1}3}}, \end{array}$$ (5)

originally adapted from the MC proposed by Mahajan et al. [22]. The other three parameters, *x*_2_, *x*_3_, and *x*_4_, multiply, respectively, the calcium-dependent function *f* presented in the rates *C* → *I*_*f*_, $C^{\prime }\to I_{f}^{\prime }$, and $O\to I_{f}^{\prime }$.

On the other hand, the voltage-dependence appears in all MC states and rates. The top layer, states *I*_*f*_ and $I_{f}^{\prime }$, and the fitting parameters *x*_5_ and *x*_6_ are associated with the voltage dependence behavior of the HH gate *f*; the bottom layer, states *I*_*f*2_ and $I_{f2}^{\prime }$, and the fitting parameters *x*_9_ and *x*_10_ are associated with the voltage dependence behavior of the HH gate *f*_2_; and the main layer, states *C*, *C*′, and *O*, and the fitting parameters *x*_7_ and *x*_8_ are associated with the voltage dependence behavior of the HH gate *d*. Concerning the fitting process, *x*_5_ and *x*_6_ are used to fit the voltage dependence (top layer); the parameters *x*_9_ and *x*_10_ are used to fit the bottom layer, and *x*_7_ and *x*_8_ are used to fit the main layer. To see a more detailed description of the new MC model, please see S1 Appendix.

### Fitting algorithm

In the above section, we propose a MC-based model to use in the I_CaL_ formulation for both Ten Tusscher and Panfilov [23], and Stewart et al. [24] models replacing the gate-based opening fraction equations. Next, it is necessary to adjust the MC fitting parameters set ***x*** to reproduce the original outputs.

The fitting procedure has one primary objective: to find the eleven parameters *x*_*i*_ that make the Opening fraction of the new MC-based model able to reproduce the original Gate-based opening values. As we are only replacing the I_CaL_ opening fraction, once we recover its dynamics, we also recover the I_CaL_ current and, consequently, all the other model outputs, such as the AP and intracellular calcium.

As can be seen in Eq (2), for both models, TP, and ST, the multiplication *dff*_2_ *f*_*cass*_ determines the opening fraction, *O*. The curves generated by the two respective models are shown in Fig 1A.

The goal is to replace these opening fraction terms by our proposed MC-based open state and to find a parameter set ***x*** capable to recover the original model outputs. So, we can see it as a minimization problem where we want to find the best set of parameters ***x*** that minimizes the error between the new MC-based I_CaL_ curve and the original one. As a minimization problem, we have to chose the objective function, or fitness function, *F*. We defined as target as the I_CaL_ curve of the eleventh pulse (after a series of stimulated AP pulses). So, for the individual, or candidate set ***x***, the *F* function reads

$$\begin{array}{c} F\left(x\right)=\sqrt{\sum_{t=10s}^{11s}\frac{{\left(I_{CaL_{MC}}\left(x,t\right)-\bar{I_{CaL_{HH}}}\left(t\right)\right)}^{2}}{\bar{I_{CaL_{HH}}}{\left(t\right)}^{2}}}, \end{array}$$ (6)

where $\bar{I_{CaL_{HH}}}$ is the calcium current of the HH gate-based model and the $I_{CaL_{MC}}$ is the calcium current generated by the new MC-based model using the parameter set ***x***. When we adjust the MC-based model to recover the TP model, our target is $\bar{I_{CaL_{HH}}}=\bar{I_{CaL_{TP}}}$. When adjusting to the ST model, our target is $\bar{I_{CaL_{HH}}}=\bar{I_{CaL_{ST}}}$. To obtain the I_CaL_ curves for the models, we simulated them using a pacing of 1Hz. We must point out, however, that we have checked the robustness of our approach to different pacing frequencies. Our best fits reproduce accurately the target data for different frequencies. These results can be found in the S2 Appendix.

To solve this optimization problem, we used the Differential Evolution (DE) algorithm available in the Python library Pygmo [27]. In the DE field, each set of parameters ***x*** is labeled as an individual or possible solution. Moreover, a set of individuals, or solutions, is labeled as population. The primary purpose of an evolutionary algorithm as DE is to begin from a random initial population and, generations over generations, to evolve this population to find individuals, or solutions, that better solve the minimization problem. For more details about the DE algorithm, please see [28].

To execute the DE, we set the population size as 100 individuals, and, to generate the initial population used by the algorithm, we used the Latin Hypercube method available in Python library SMT [29]. We set the number of generations of the algorithm to 50. So, at the end of the DE execution, we will have 50 × 100 possible solutions. The optimization algorithm is constrained by imposing limits for each one of the parameter. The search space S for the parameters was set as $S=\{S\subset {\mathbb{R}}^{11}|0.1\leq s_{i}\leq 5.9\}$, where *s*_*i*_ is the search space of the respective parameter *x*_*i*_. All the other algorithm settings were adopted as the standard values implemented by the Pygmo library. To see more details about its settings, please see [27]. The cardiac models were implemented in C++. To simulate them, we used a multistep numerical method provided by C++ SUNDIALS CVODE library [30] setting the maximum time step as 10^−1^ms.

### Fitting robustness assessment

To analyze the fitting process of the new MC-based model, we considered a collection of the individuals found by the Differential Evolution algorithm, labeled as population of solutions. To compose this collection, or population, *P*_•_ of the best solutions, we took into account how each solution contributes to the respective population. After sorting all the 5000 solutions from the best error *F* up to the worst, we could see that the relation between the individuals and their respective error could be seen as two different relations. The first one is rather a linear relation and, the second one is similar to an exponential relation. In this way, we concluded that the solutions in the exponential fraction should be discarded since the error they bring is higher than the possible quality it would aggregate.

Then, considering only the linear fraction of the ratio, we could select as many solutions as we could compute. At this point, we might also consider the computational cost. So, looking at the computational cost and driven by the acknowledgment of model discrepancy and the biological variability usually found in the experiments, 10 − 20%, from all 5000 possibilities obtained by the DE for each target model, we selected the best 300 solutions considering the fitness function error *F*, which was equivalent to including all solutions that satisfy *F*(***x***)≤16%, or 6% of the solutions (300/5000). S1 Fig in the Supporting Information presents the ratio between the sorted individuals and their respective errors *F*.

For each target model, TP and, ST, we generated the respective population *P*_*TP*_, and *P*_*ST*_. Furthermore, in the results, we highlight the best solution of the respective populations, *P*_*TP*_, and *P*_*ST*_, labeled as $x_{TP}^{b}$, and $x_{ST}^{b}$. It means the best solution which obtained the smallest error $F(x_{•}^{b})$ for each respective model.

Besides the statistical analysis, we also assess the robustness of the fitting process by evaluating if the same population of solutions *P*_•_ can reproduce the original model (target data) under a modified protocol. For that, we chose a protocol where the Calcium Inactivation is suppressed. This protocol simulates the condition of Calmodulin mutations associated with long QT syndrome which is known to promote proarrhythmic behavior in ventricular myocytes [31].

**Fig 3:**
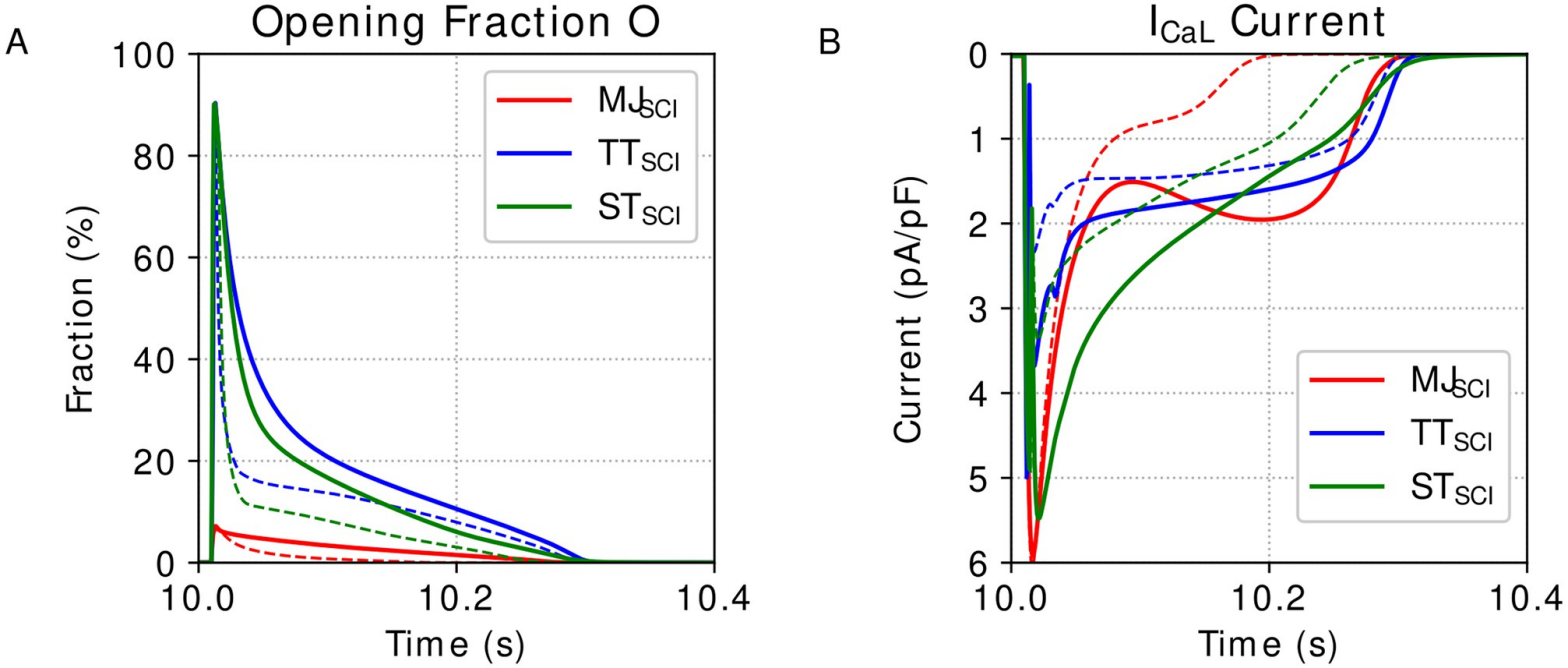
Cardiac models outputs under SCI protocol. Output dynamics of the models Mahajan et al. [22] (MJ), Ten Tusscher and Panfilov [23] (TP), and Stewart et al. [24] (ST) under Suppressed Calcium Inactivation (SCI) protocol. The dashed lines represent the same variables but in full protocol. A: Opening Fraction, *O*. B: I_CaL_ current.

This protocol, Suppressed Calcium Inactivation (SCI), consists of removing the calcium sensitivity inside the Gate-based models and the MC-based models. To simulate this SCI protocol in the TP and in the ST original models, we set the gate *f*_*cass*_ equals 1. To simulate the SCI protocol in the MC-based formulation, we will set the calcium level *c* inside the function *f* = *f*(*x*_0_, *x*_1_, *c*), Eq (5), as constant. Fig 3 shows the opening fraction, *O*, and I_CaL_ curves for the models Mahajan et al. [22], Ten Tusscher and Panfilov [23], and Stewart et al. [24] under the SCI protocol. Considering the protocol SCI, we evaluate how the population of models *P*_•_, which was fitted using a different protocol (Full), can reproduce the experiments TP|_SCI_, and ST|_SCI_.

Although we only use the fitness function *F*(***x***) to select the parameters of the models, we also evaluate the solutions using some important biomarkers for the AP and the Intracellular Calcium concentration ([*Ca*]_*i*_). For the AP, we consider the features: duration from the AP peak up to 50% of decay, *APD*_50_, and the duration from the AP peak up to 90% of decay, *APD*_90_. For the [*Ca*]_*i*_ curve, the features are the Calcium basal level [*Ca*]_*i*__*min*_, and the Calcium Peak value, [*Ca*]_*i*__*Peak*_.

### Multi-protocol analysis

We also evaluate the benefits from selecting a new population of 300 solutions, from the same original 5000 candidates, that takes into account both the original fitness function *F*(***x***) as well as a similar error function for the SCI protocol. The idea behind this exercise is to evaluate how the re-sampling of a new population of solutions can accommodate new experimental evidence.

Although we did not use a multi-objective function in any case of the DE executions, we also evaluated the capacity of all the 5000 solutions described in Section Fitting algorithm to reproduce the respective original models, TP, and ST, simulated under the SCI protocol. For each solution ***x***, now, we have two errors associated with it: the Full protocol error, *F*(***x***)|_*N*_, and the SCI protocol error, *F*(***x***)|_*SCI*_. Both, *F*(*x*)|_*N*_, and *F*(*x*)|_*SCI*_ reflects the same mathematical equations presented in Eq (6), but each one considers the models simulated under the Full, and under the SCI protocols, respectively. At this moment, we can define a new population *PO*_•_, which is composed of the best Overall solutions evaluated considering the function *OF*(***x***):

$$\begin{array}{c} OF\left(x\right)=\sqrt{F^{2}\left(x\right){{|}_{N}+F^{2}\left(x\right)|}_{SCI}}. \end{array}$$ (7)

This new populations *PO*_*TP*_, and *PO*_*ST*_, obtained for the models TP, and ST, bring their respective new best solutions $x_{TP}^{o}$ and $x_{ST}^{o}$.

In summary, we will use a color scheme to represent the main findings in Results section. The black markers presents the original TP, and ST models; the blue markers present the selected MC-based solutions which compose the population of solutions found for the TP model, *P*_*TP*_; the best solution of the population *P*_*TP*_, $x_{TP}^{b}$, is highlighted using the dark blues color; the red markers present the selected MC-based solutions which compose the population of solutions found for the ST model, *P*_*ST*_; the best solution of the population *P*_*ST*_, $x_{ST}^{b}$, is highlighted using the dark red color; the orange markers present the selected MC-based solutions which compose the population of the best overall solutions found for the TP model, *PO*_*TP*_; the best solution of the population *PO*_*TP*_, $x_{TP}^{o}$, is highlighted using the dark orange color; the green markers present the selected MC-based solutions which compose the population of the best overall solutions found for the ST model, *PO*_*ST*_; the best solution of the population *PO*_*ST*_, $x_{ST}^{o}$, is highlighted using the dark green color. The same scheme of colors are used to show the results for both, Full, and SCI protocols. Finally, the notation •|_*SCI*_ represents the simulation under the SCI protocol.

### Statistical and sensitivity analysis

The fitting process involves a set of parameters and objective targets. However, as soon as the size of the parameter set increases, it becomes more complicated to understand the correlation between each parameter and the target outputs. Sensitivity Analysis is a tool that improves this understanding. This process consists of checking how each parameter influences the outputs.

In this study, we perform a Sensitivity Analysis based on variances to understand how each one of the 11 parameters (*x*_0_ up to *x*_10_) influences the fitness error *F*(***x***). For that, we calculate the 1^*st*^-order, and the Total-order Sobol Sensitivity Index. The 1^*st*^-order sensitivity index quantifies only the portion that an input parameter contributes directly to the total variance of the quantity of interest. The Total-order index also considers the sensitivity generated by the interaction between the parameters. For more details see [32]. To calculate these sensitivity indexes, we used the Python library ChaosPy [33].

## Results

### Successful fitting of the new markov chain-based under full protocol—Training

We proceed to show how the MC-framework explained in Section Markov chain-based model can properly fit the output of the two models selected as guiding examples in our study: Ten Tusscher and Panfilov [23] (TP model), and Stewart et al. [24] (ST model).

**Fig 4:**
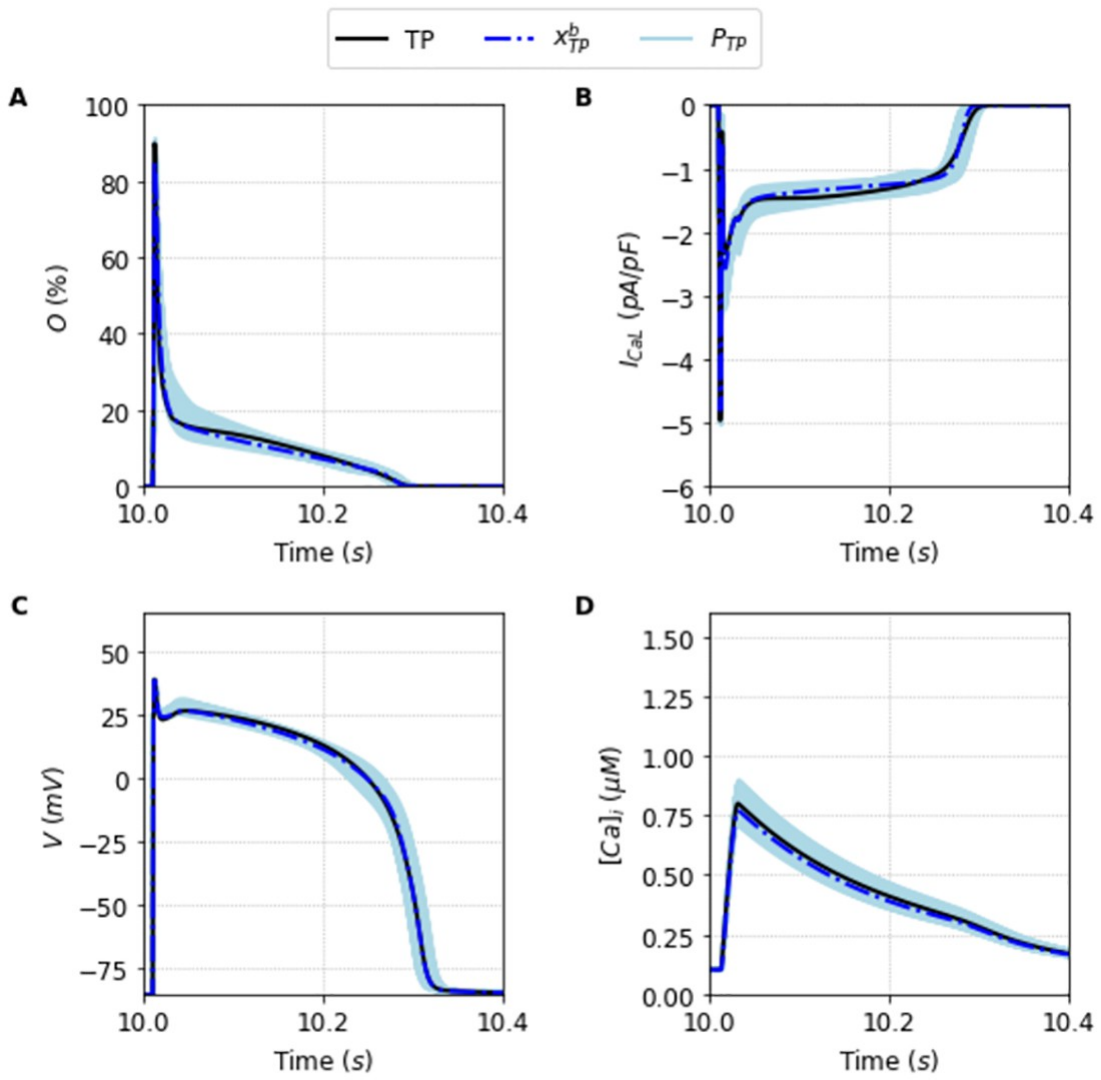
Outputs generated by the population of solutions *P*_*TP*_ simulated under the Full protocol. Traces obtained using the best solution of the population *P*_*TP*_, $x_{TP}^{b}$ (dashed blue line), alongside the traces obtained using the other solutions that compose the population (light blue lines) compared with the original Ten Tusscher and Panfilov [23] model (black line) simulated under the Full protocol. A: Opening fraction, *O*. B: I_CaL_ current. C: Action Potential. D: Intracellular Calcium concentration, [*Ca*]_*i*_.

**Fig 5:**
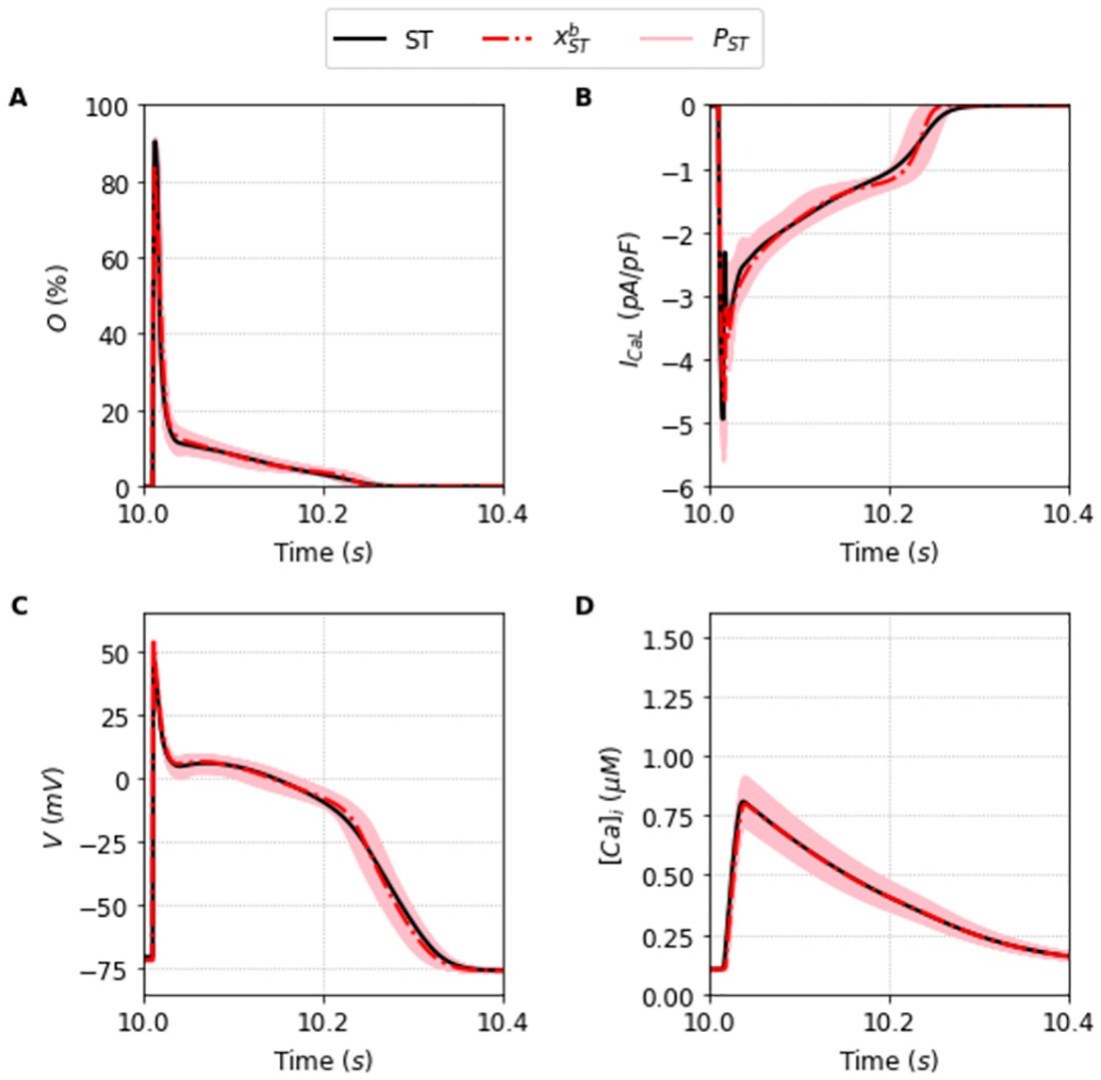
Outputs generated by the population of solutions *P*_*ST*_ simulated under the Full protocol. Traces obtained using the best solution of the population *P*_*ST*_, $x_{ST}^{b}$ (dashed red line), alongside the traces obtained using the other solutions of the population (light red lines) compared with the original Stewart et al. [24] model (black line) simulated under the Full protocol. A: Opening fraction, *O*. B: I_CaL_ current. C: Action Potential. D: Intracellular Calcium concentration, [*Ca*]_*i*_.

We perform a single execution of the DE algorithm (see Section Fitting algorithm) for each of the two target models. We plot in Figs 4 and 5 the original traces for the Opening fraction of the LCC, the I_CaL_ current, the AP and the Calcium transient [*Ca*]_*i*_ for the TP and the ST model respectively. In each panel, the original output (black line) is plotted together with the results of the population selection of the algorithm together with the best global fit of this population (blue lines for the TP model, and red lines for the ST model). The parameter values obtained from the optimization correctly reproduce the action potentials under normal (1 Hz) and faster pacings. See S2 Appendix.

The selection of the population made by the DE execution is very good for both TP, and ST cases. The key is not that there is a particular solution that fits almost perfectly the outcome, but that the population spans remarkably the general surroundings of the model-space. We can see in Table 1 a comparison of the key properties of the AP and [*Ca*]_*i*_ for each original model with the average and standard deviation values obtained from the selected populations *P*_•_. These populations of solutions *P*_•_ found under the Full protocol are perfectly fit with typical errors around 0–1% except for the error in the peak of calcium of the ST model where it reaches values close to 4%.

**Table 1:** Features for the AP and [*Ca*]_*i*_ traces obtained by the simulations under Full protocol.

Full Protocol						
Features	TP Model			ST Model		
	*P*_*TP*_	TP	MAPE^a^	*P*_*ST*_	ST	MAPE^a^
*APD*_50_ ^b^	275.2 ± 4.9	273	1.62%	197.6 ± 9.3	195	4.03%
*APD*_90_ ^b^	301.8 ± 4.8	301	1.33%	286.5 ± 7.0	292	2.53%
[*Ca*]_*i*__*Min*_ ^c^	0.1 ± 0.001	0.1	1.04%	0.1 ± 0.002	0.1	1.86%
[*Ca*]_*i*__*Peak*_ ^c^	0.8 ± 0.04	0.81	4.02%	0.79 ± 0.04	0.81	5.09%

Values of the features for the Action Potential (AP) and Intracellular Calcium concentration ([*Ca*]_*i*_) for the respective populations of solutions *P*_*TP*_ found for the TP model [23], and *P*_*ST*_ found for the ST model [24] in comparison with respective original models values simulated under Full protocol.

^a^The errors are expressed as the Mean Absolute Percentage Error (MAPE) of the population. It can be computed as $\text{MAPE}=\frac{100}{n}\sum^{x}|{\text{Feature}}_{x}-{\text{Feature}}_{m}|/{\text{Feature}}_{m}$, where Feature_*x*_ is the value of the feature obtained using the solution *x*, and Feature_*m*_ is the value of the same feature obtained simulating the respective original model *m*. The summation is calculated over all the *n* solutions that compose the population *P*_•_.

^b^Values are expressed in *ms*.

^c^Values are expressed in *μM*.

Another important fact is that the values achieved by the objective fitness function to minimize Eq (6) are not close to zero. The best parameter fit for each model was $F(x_{TP}^{b})=9\%$ and $F(x_{ST}^{b})=13\%$ for the TP and ST model, respectively. This indicates that the best parameters cannot exactly reproduce the Full I_CaL_ current. This is a typical case of model discrepancy, as described before in [34]. In this case, a good practice is to accept solutions with a higher tolerance for the fitness value. In this direction, the average value of the objective function of the I_CaL_ current obtained by the solutions that compose the population *P*_*TP*_ is around *F* = 13% ± 1, and *F* = 15% ± 1 for the solutions that compose the population *P*_*ST*_. It is the whole population that is capable of providing a good ensemble. They are good enough to be in the population as they reasonably fit the model outcome and, as we will see, give us the flexibility to fit intercellular or intermodel differences in the outcome. We proceed to show its usefulness discussing first the limitation of taking only the best fit, $x_{•}^{b}$.

### Limitation of the best fit in a protocol with suppression of the calcium inactivation in the LCC

We proceed now to implement a protocol where we suppress all calcium dependence in the LCCs. Now, the two models produce not only different I_CaL_ currents but also slightly different APD and very different calcium transient given the well-known effects of calcium transient in the inactivation of the I_CaL_ current. More specifically, the output gives larger currents and larger calcium transients with the new protocol. The aim of this suppression of the calcium inactivation (SCI protocol) is to mimic the results of an pathological condition associated with proarrhythmic behavior. There were many options available but we think that a short-circuit of the calcium inactivation of the LCC is a perfect computational example of a pseudo-experiment.

We can now compare how the average population behaves with the new protocol. We check how our former best parameter fit solution, that we call $x_{TP}^{b}$ for the TP model and $x_{ST}^{b}$ for the ST model, fits the outcome of the new APD and calcium transients under the new protocol that suppresses calcium inactivation of the LCC. Table 2 shows the performance of the average of the population for respective models for the same benchmarks used in the Full protocol. We can see how the average errors are larger. This makes perfect sense, since the population *P*_•_ was obtained using a different protocol (Full protocol). However, it is quite impressive that these errors are not very large for APD and the minimum calcium level. The most relevant differences appear in the calcium peak, where typical 20–30% errors are present. The reason is clear: differences in the I_CaL_ current do not affect the APD if they are not rather large, given the presence of other currents that do not provide a significant feedback into the voltage-dependence nature of the LCC. On the other hand, there is a strong feedback between the transient of calcium and the inactivation of the LCC, precisely because of the Calcium-Induced Calcium-Release nature of the calcium release trigger.

**Table 2:** Features for the AP and [*Ca*]_*i*_ traces obtained by the simulations under the SCI protocol.

SCI Protocol						
Features	TP Model			ST Model		
	*P*_*TP*_|_*SCI*_	TP|_SCI_	MAPE^a^	*P*_*ST*_|_*SCI*_	ST|_SCI_	MAPE^a^
*APD*_50_ ^b^	279.9 ± 5.4	282	1.62%	221.4 ± 29.1	248	13.56%
*APD*_90_ ^b^	306.6 ± 5.3	311	1.78%	308.8 ± 28.3	343	11.52%
[*Ca*]_*i*__*Min*_ ^c^	0.11 ± 0.002	0.11	5.47%	0.11 ± 0.006	0.12	9.74%
[*Ca*]_*i*__*Peak*_ ^c^	0.86 ± 0.05	1.08	20.05%	1.0 ± 0.28	1.45	33.81%

Values of the features for the Action Potential (AP) and Intracellular Calcium concentration ([*Ca*]_*i*_) for the respective populations of solutions *P*_*TP*_ found for the TP model [23], and *P*_*ST*_ found for the ST model [24] in comparison with respective original models values simulated under SCI protocol.

^a^The errors are expressed as the Mean Absolute Percentage Error (MAPE) of the population. It can be computed as $\text{MAPE}=\frac{100}{n}\sum^{x}|{\text{Feature}}_{x}-{\text{Feature}}_{m}|/{\text{Feature}}_{m}$, where Feature_*x*_ is the value of the feature obtained using the solution *x*, and Feature_*m*_ is the value of the same feature obtained simulating the respective original model *m*. The summation is calculated over all the *n* solutions that compose the population *P*_•_.

^b^Values are expressed in *ms*.

^c^Values are expressed in *μM*.

In the SCI case, the population of solutions *P*_*TP*_|_*SCI*_ gave an error of roughly 21% ± 3, while the population of solutions *P*_*ST*_|_*SCI*_ obtained and error of 29% ± 7 when compared with the respective original TP|_SCI_, and ST|_SCI_ under the same conditions. So we can easily see that the errors are more prominent for the SCI protocol when compared to the control or full protocol. We must emphasize again that we are not finding a new population to fit the new protocol data. We are just testing how the population we found in the previous section behaves when the inactivation dependence of the LCC is suppressed.

**Fig 6:**
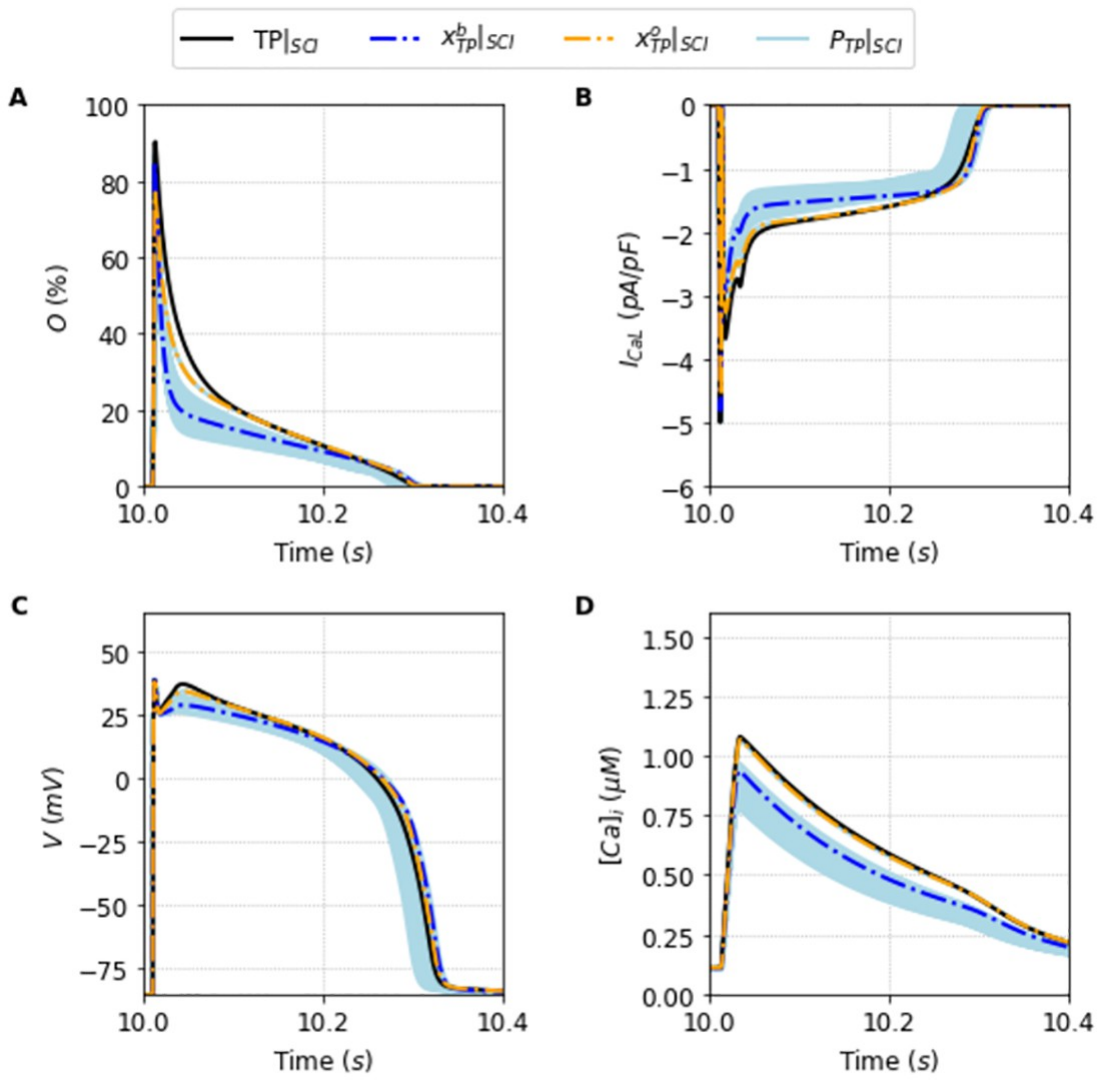
Outputs generated by the population of solutions *P*_*TP*_ simulated under the SCI protocol. Traces of the best solution of the population *P*_*TP*_ simulated under SCI protocol, $x_{TP}^{b}$ (dashed blue line), and the best overall solution $x_{TP}^{o}$ (dashed orange line) simulated under SCI protocol, alongside the traces generated by the simulation of the population of solutions *P*_*TP*_|_*SCI*_ (light blue lines) compared with the original Ten Tusscher and Panfilov [23] model (black line) under the SCI protocol. A: Opening fraction, *O*. B: I_CaL_ current. C: Action Potential. D: Intracellular Calcium concentration, [*Ca*]_*i*_.

**Fig 7:**
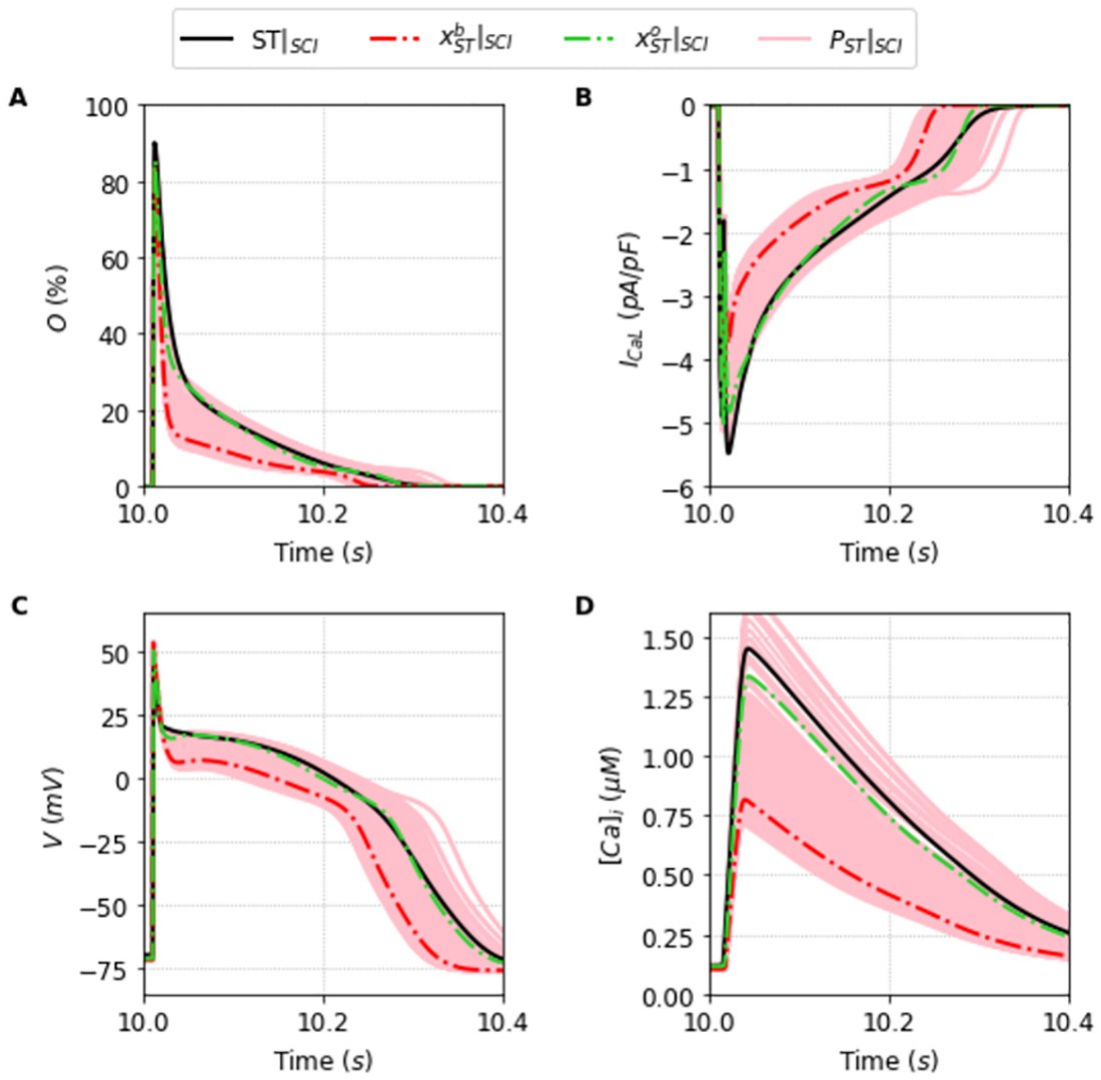
Outputs generated by the population of solutions *P*_*ST*_ simulated under the SCI protocol. Traces of the best solution of the population *P*_*ST*_ simulated under SCI protocol, $x_{ST}^{b}$ (dashed red line), and the best overall solution $x_{ST}^{o}$ (dashed green line) simulated under SCI protocol found for the [24] model, alongside the traces generated by the simulation of the population of solutions *P*_*ST*_|_*SCI*_ (light red lines) compared with the original Stewart et al. [24] model (black line) under the SCI protocol. A: Opening fraction, *O*. B: I_CaL_ current. C: Action Potential. D: Intracellular Calcium concentration, [*Ca*]_*i*_.

In this sense, Figs 6 and 7 are particularly relevant. They show, respectively, the new traces of the outputs generated using the population of solutions *P*_*TP*_|_*SCI*_ compared to the model TP|_SCI_ TP|_SCI_, and the new traces of the outputs generated using the population *P*_*ST*_|_*SCI*_ compared with the model ST|_SCI_. The blue dashed lines, and the red dashed lines highlight how the best solutions, $x_{TP}^{b}{|}_{SCI}$, and $x_{ST}^{b}{|}_{SCI}$ are clearly not the best solutions of each respective populations now.

We can observe in Figs 6 and 7 the relevance of having a broad population that can encompass different experimental data or different protocol data. The population in the new protocol generally shifts on average away from the new outcome, but it is broad enough, as we will see now, to be able to find a new best fit solution that encompasses the new input data.

### Robustness of the population of solutions—generalization

We proceed to analyze whether the original populations *P*_•_ were flexible enough to find a set of parameters that can properly fit both the initial data and the new data obtained with the SCI protocol. In the previous section we have shown how the best solutions for the models outcome are not the most robust solutions under the addition of data from a new protocol. To select the best overall solution for each target model, we considered the overall fit *OF*(***x***) as described in Eq (7).

We calculate this overall fit for all the solutions that were already present in the population selected in Section Successful fitting of the new markov chain-based under full protocol—training. This is an important point. We do not need to go back and re-obtain a population around this new minimization with a new objective function. If the population is robust and broad enough, analyzing it should provide a reasonably good solution with a small overall error. This is indeed the case. The new best solutions, $x_{TP}^{o}$, and $x_{ST}^{o}$, are plotted respectively as a orange dashed line in Fig 6, and as a green dashed line in Fig 7. It is remarkably good.

**Fig 8:**
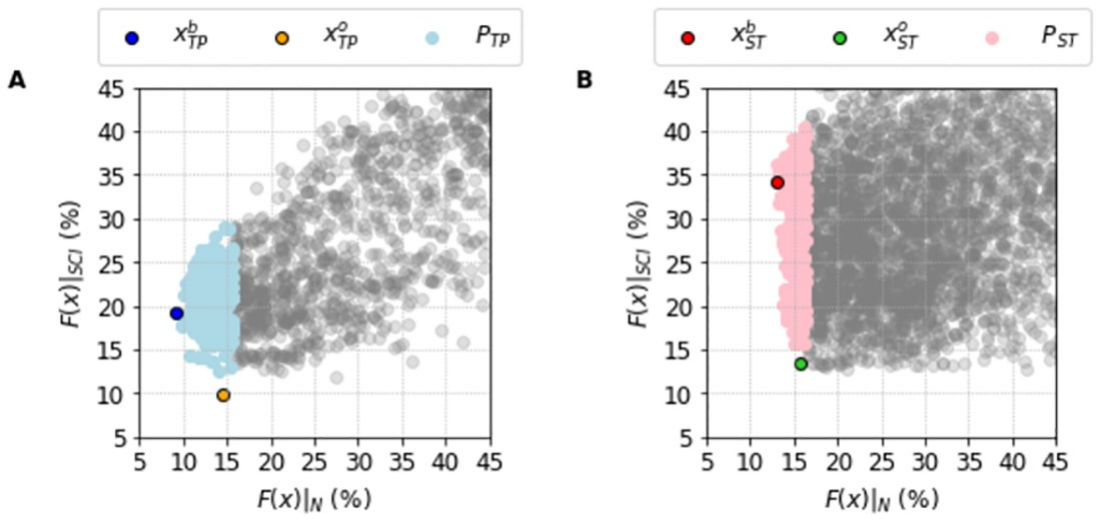
Correlation between the Full and SCI protocols errors of the population of solutions *P*_•_. Values of the Full protocol and SCI protocols errors, respectively *F*(***x***)|_*N*_ and *F*(***x***)|_*SCI*_. A: The best Full protocol solution, $x_{TP}^{b}$ (blue dot), the best Overall solution $x_{TP}^{o}$ (orange dot) alongside all the solutions that compose the population *P*_*TP*_ (light blue dots) found for the Ten Tusscher and Panfilov [23] model. B: The best Full protocol solution, $x_{ST}^{b}$ (red dot), the best Overall solution $x_{ST}^{o}$ (green dot) alongside all the solutions that compose the population *P*_*ST*_ (light red dots) found for the Stewart et al. [24] model. The other representations (gray dots) are the solutions that became out of the respective population *P*_•_.

Fig 8 shows the idea behind this selection. We do a scatter plot where each solution in the population is placed with its two fitness functions, one for the normal output (X-Axis) and one for the SCI protocol (Y-Axis). We can see the cloud of points indicating how the population properly spans reasonable errors. However, the best fit of the full protocol (blue dot for the TP Model, and red dot for the ST Model) are not the best overall fit (orange dot for the TP Model, and green dot for the ST Model). For example, in the TP model the best overall fit has a value of $OF(x_{TP}^{o})=18\%$ with $F(x_{TP}^{o}){|}_{N}$ around 15% and $F(x_{TP}^{o}){|}_{SCI}$ around 10%. This is a clear improvement from the best solution when the new protocol was not taken into consideration. The $x_{TP}^{b}$ solution has $F(x_{TP}^{b}){|}_{N}$ at 9% as indicated previously, but with the new protocol $F(x_{TP}^{b}){|}_{SCI}$ is around 20%.

Remarkably, we observe exactly the same structure for the population obtained for the ST data/model. We again find that the population is very robust and can produce an overall best fit without the need of retraining. In this model, the penalty we encounter in order to move from the original model output to the inclusion of the new protocol is way smaller than in the TP model but we cannot achieve the same very low values of *F*(***x***)|_*SCI*_.

The clouds presented in Fig 8 also show that the selected populations of solutions *P*_•_ have a narrow range for *F*(***x***)|_*N*_, but spans a wider range for *F*(***x***)|_*SCI*_. This clearly reflects that the individuals of the populations *P*_•_ were selected to account only for the smallest values of *F*(***x***)|_*N*_.

**Fig 9:**
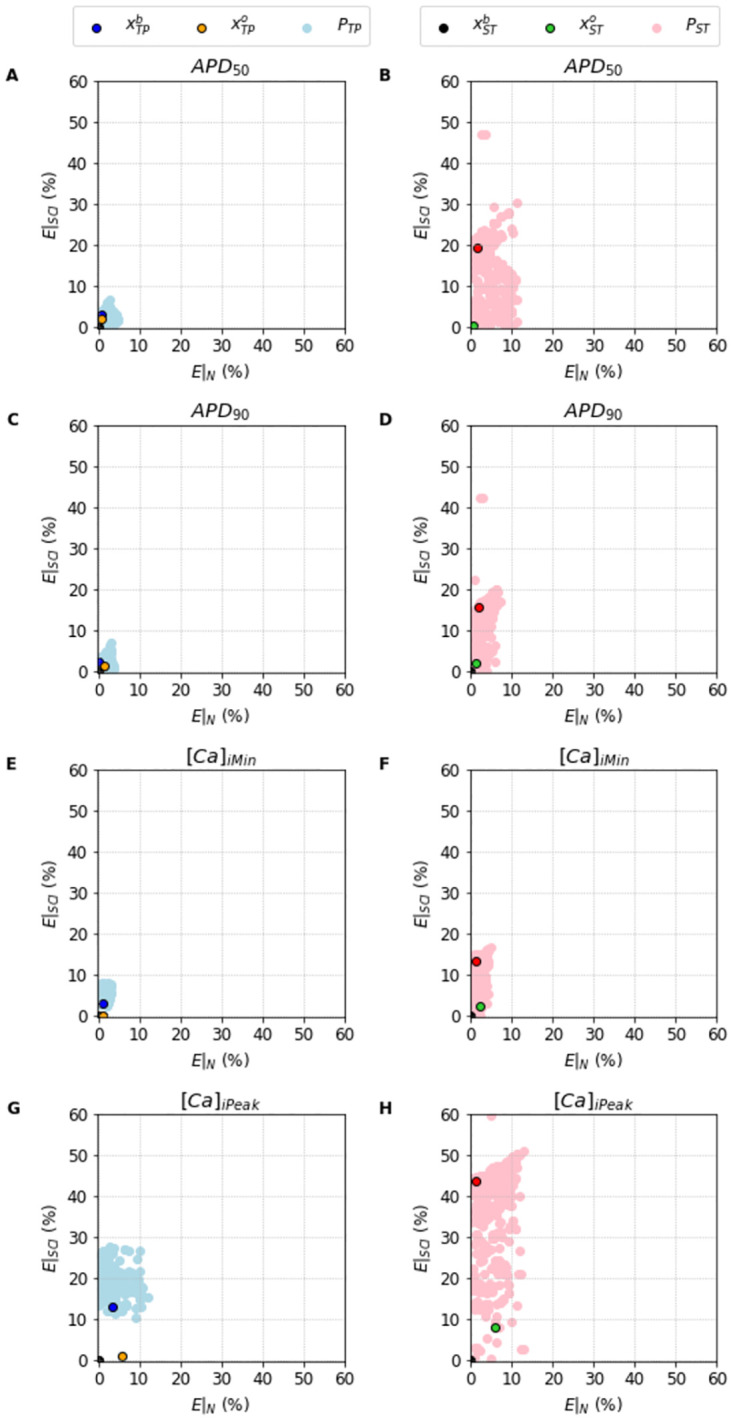
Errors of the features of the AP and [*Ca*]_*i*_ traces obtained by the population of solutions *P*_•_. The left column shows the population of feasible solutions *P*_*TP*_ (light blue dots) found for the Ten Tusscher and Panfilov [23], and highlights the best solution $x_{TP}^{b}$ (blue dot) alongside the best Overall solution $x_{TP}^{o}$ (orange dots) found for the same model. The right column shows the population of feasible solutions *P*_*ST*_ (gray dots) found for the Stewart et al. [24], and highlights the best solution $x_{ST}^{b}$ (red dot) alongside the best Overall solution $x_{ST}^{o}$ (green dots) found for the same model. A: TP Model *APD*_50_. B: ST Model *APD*_50_. C: TP Model *APD*_90_. D: ST Model *APD*_90_. E: TP Model [*Ca*]_*i*__*Min*_. F: ST Model [*Ca*]_*i*__*Min*_. G: TP Model [*Ca*]_*i*__*Peak*_. H: ST Model [*Ca*]_*i*__*Peak*_. The errors were calculated using *E*|_•_(***x***) = |Feature_***x***_−Feature_*m*_|/Feature_*m*_, where Feature_***x***_ is the value of the respective features obtained simulating the solution ***x***, and Feature_*m*_ is the value of the respective features obtained simulating the original models (or target models).

The characteristics of the population can be better understood in Fig 9 where we highlight the different properties of the clouds in terms of the errors in AP and [*Ca*]_*i*_ features for both models. Once again, the clouds occupy more space in the upper side of the SCI protocol since the objective function was to minimize the distance to the I_CaL_ current between the model output and our MC-based model. Nevertheless, we see that our population is flexible and robust enough to find a subset of solutions that clearly manage to provide good values for the benchmarks. Indeed, the best overall fits provide rather low errors for I_CaL_, AP and [*Ca*]_*i*_. In addition, once we have the populations selected, we might focus on the I_CaL_ current to obtain the best overall fit as we have done until now and shown in Figs 6–9, or we can focus on any other particular feature that we might find more important or relevant, whether it is I_CaL_, AP or [*Ca*]_*i*_ related.

### Learning from new data—Sensitivity analysis, identifiability, and multi-objective function

**Fig 10:**
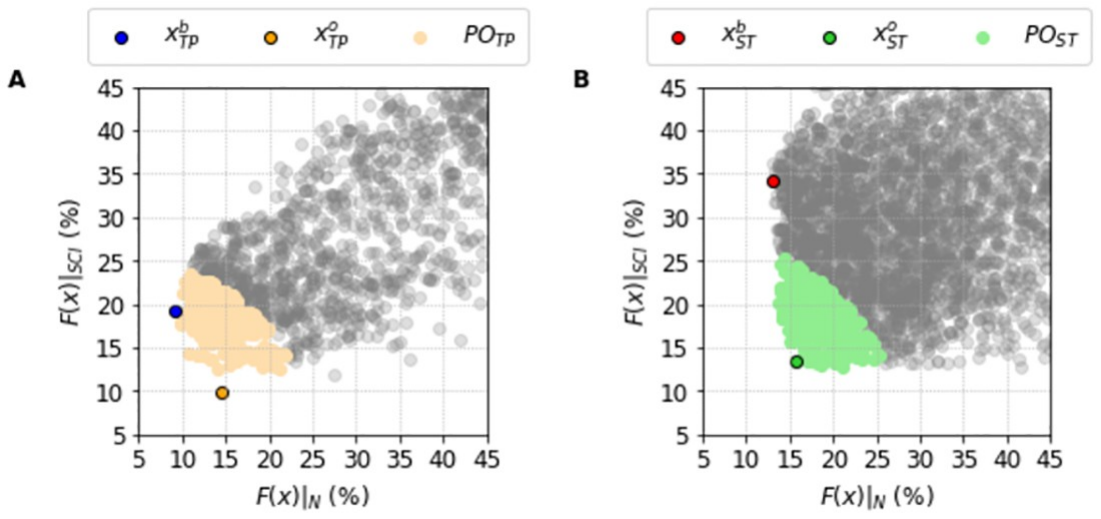
Correlation between the Full and SCI protocols errors of the population of solutions *PO*_•_. Values of the Full and SCI protocols errors, respectively *F*(***x***)|_*N*_ and *F*(***x***)|_*SCI*_. A: The best Full protocol solution $x_{TP}^{b}$ (blue dot), the best Overall solution $x_{TP}^{o}$ (orange dot) alongside all the solutions that compose the population *PO*_*TP*_ (light orange dots) selected for the Ten Tusscher and Panfilov [23] model. B: The best Full protocol solution $x_{ST}^{b}$ (red dot), the best Overall solution $x_{ST}^{o}$ (green dot) alongside all the solutions that compose the population *PO*_*ST*_ (light green dots) selected for the Stewart et al. [24] model. The other representations (gray dots) are the solutions that became out of the respective populations *PO*_•_.

**Fig 11:**
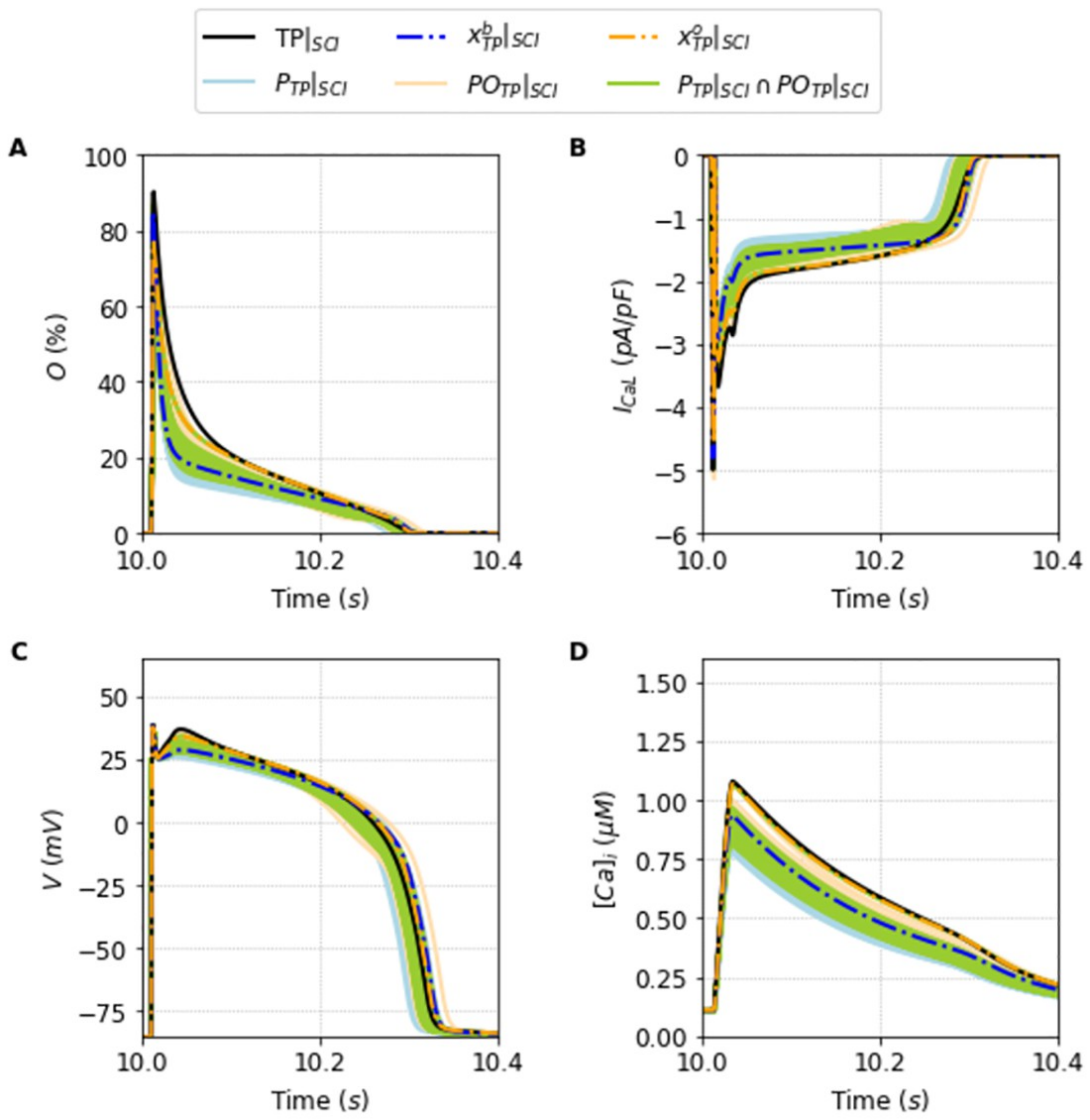
Traces of the populations of fitting solutions *P*_*TP*_ and *PO*_*TP*_ under SCI protocol. The light blue lines are the 300 best solutions considering the objective function *F* which compose the population *P*_*TP*_. The light orange lines are the 300 best solutions considering the overall fit *OF* which compose the population *PO*_*TP*_. Naturally, the solutions that are present in both populations are shown as light green lines. Furthermore, the two dashed lines, the blue and the orange one, represent the best solution $x_{TP}^{b}$ and the solution $x_{TP}^{o}$ respectively. The black line represents the Ten Tusscher and Panfilov [23] model. A: Opening fraction, *O*. B: I_CaL_ current. C: Action Potential. D: Intracellular Calcium concentration [*Ca*]_*i*_.

**Fig 12:**
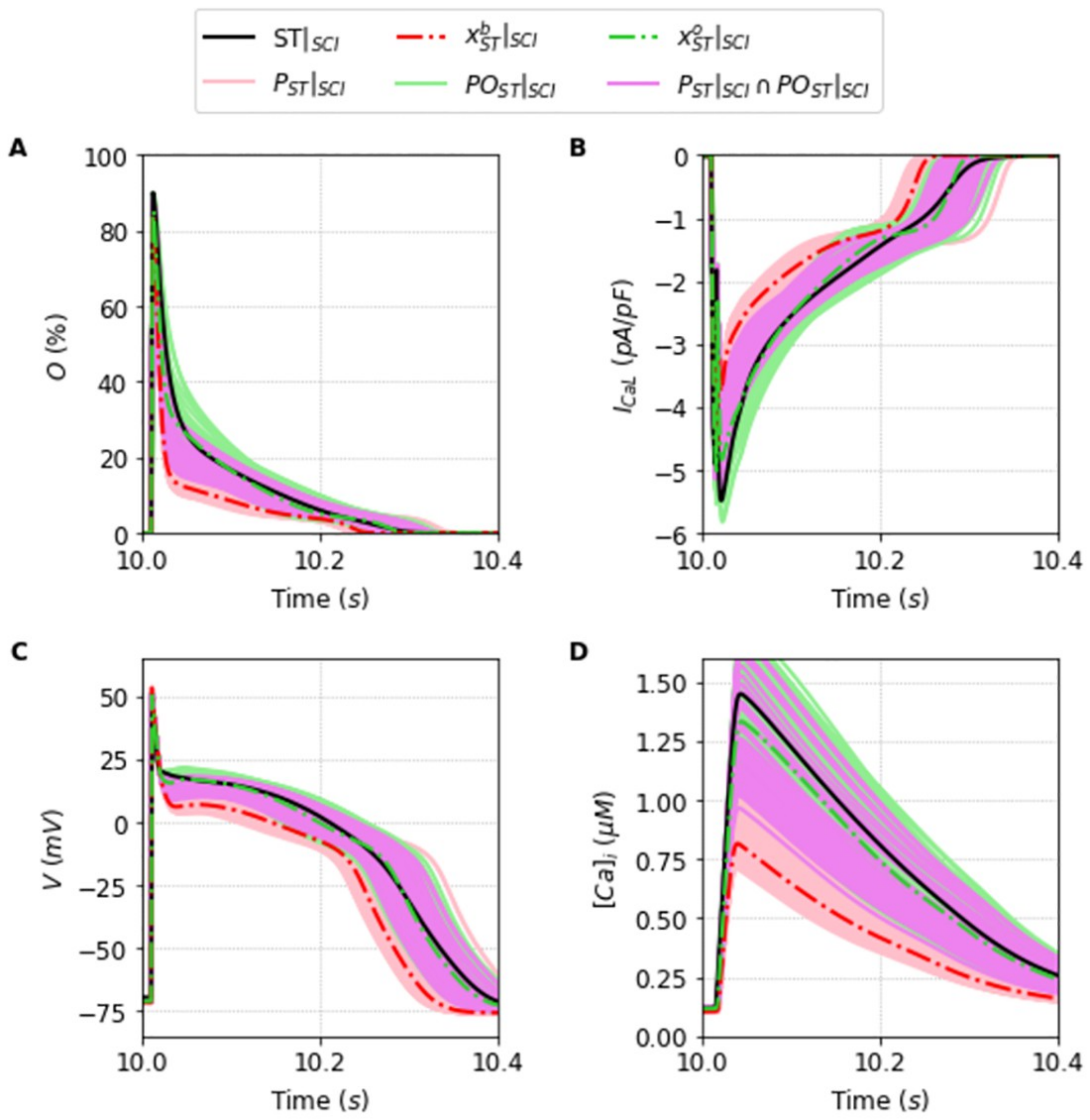
Traces of the populations of fitting solutions *P*_*ST*_ and *PO*_*ST*_ under SCI protocol. The light red lines are the 300 best solutions considering the objective function *F* which compose the population *P*_*ST*_. The light green lines are the 300 best solutions considering the overall fit *OF* which compose the population *PO*_*ST*_. Naturally, the solutions that are present in both populations are shown as purple lines. Furthermore, the two dashed lines, the red and the green one, represent the best solution $x_{ST}^{b}$ and the solution $x_{ST}^{o}$ respectively. The black line represents the Stewart et al. [24] model. A: Opening fraction, *O*. B: I_CaL_ current. C: Action Potential. D: Intracellular Calcium concentration [*Ca*]_*i*_.

So far we have performed the traditional steps of machine learning: 1) training a population of solutions; and 2) testing how it generalizes to new data (SCI protocol). Fig 10 shows the case where we assimilate the new data to create a new populations of solutions *PO*_•_, that comprises the 300 solutions with smallest overall fitness, see Eq (7). A Pareto front [35] can be easily spotted highlighting the compromise between the two different datasets. Figs 11 and 12 show how these new populations evaluated under the SCI protocol, *PO*_*TP*_|_*SCI*_, and *PO*_*ST*_|_*SCI*_, can better reproduce the two different data for the TP|_SCI_, and ST|_SCI_ cases, respectively.

**Fig 13:**
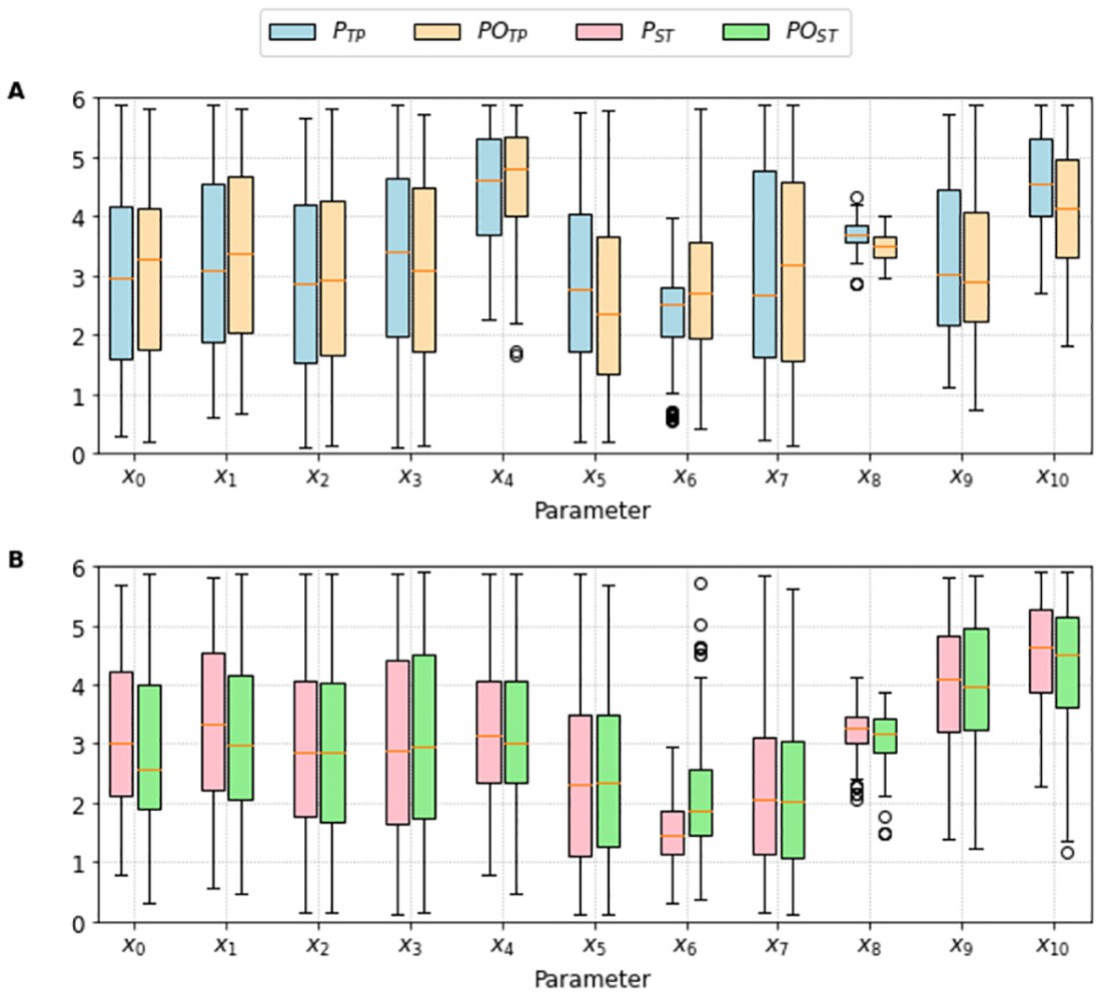
Parameter ranges of the two populations of solutions *P*_•_ and *PO*_•_. Descriptive measures of the values that each fitting parameter assumed among the best feasible solutions for each respective model. The light blue and light orange bars present, respectively, the solutions which compose the populations *P*_*TP*_ and *PO*_*TP*_. The light red and light green bars present, respectively, the solutions which compose the populations *P*_*ST*_ and *PO*_*ST*_. A: Descriptive measures obtained for the Ten Tusscher and Panfilov [23] fitting process. B: Descriptive measures obtained for the Stewart et al. [24] fitting process.

**Fig 14:**
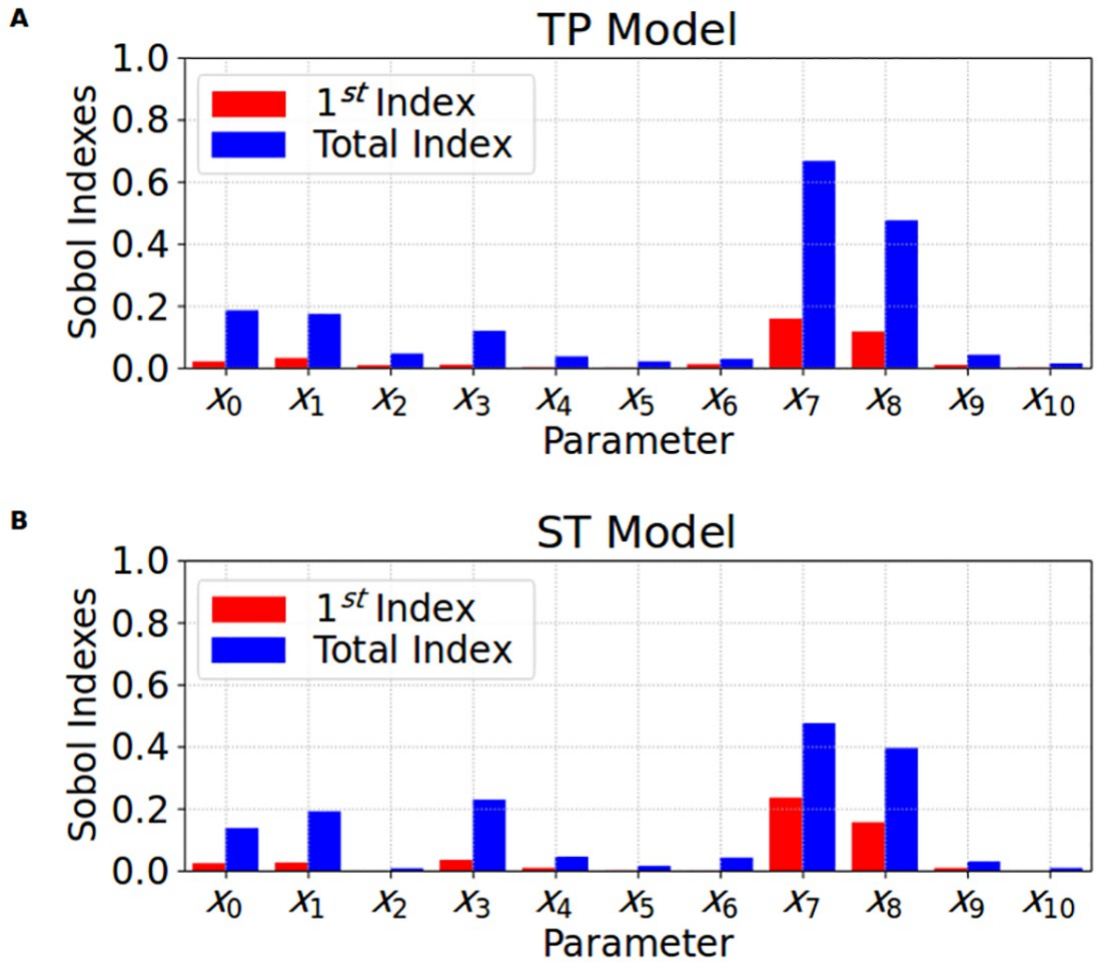
Sensitivity analysis. The Sobol 1^*st*^ (red bar), and Total (blue bar) order sensitivity indexes calculated for the 11 fitting parameters (*x*_0_ up to *x*_10_) when analyzed the influence in the fitness function *F*(***x***). A: Sensitivity indexes obtained for the Ten Tusscher and Panfilov [23] fitting process. B: Sensitivity indexes obtained for the Stewart et al. [24] fitting process.

Fig 13 presents the parameter ranges of the two populations of solutions *P*_•_ and *PO*_•_ for the TP and ST cases. Some parameters have wide ranges of values, or high variances, such as *x*_2_, *x*_3_, and *x*_7_. Others have small variances, such as *x*_6_, and *x*_8_. Furthermore, Fig 14 presents a Sensitivity Analysis done to check how each parameter influences the error function *F*(***x***). In this case, we can see how the parameter *x*_8_, besides having a small variance, also produces a high sensitivity in the MC-based model. So, these variances combined with the sensitivity analysis, can clarify how each parameter is associated with the fitting process. High variances combined with small sensitivity indices may indicate the parameters that have low influence on I_CaL_, whereas small variances combined with high sensitivity indices may highlight the most important parameters that affect the I_CaL_ dynamics.

When we move from the control populations, *P*_*TP*_ (blue bars), and *P*_*ST*_ (orange bars), to the overall populations, *PO*_*TP*_ (red bars), and *PO*_*ST*_ (green bars), we can observe what we have learned with the assimilation of new data. For instance, for parameter *x*_8_ the new data shifted its mean value and reduced its variance. In this case, the new data filtered or rejected some solutions that belonged to *P*_•_. However, the new data also expanded the original population *P*_•_ with new solutions. This is the case for instance, for parameters *x*_6_ and *x*_9_. For these, the new data shifted their mean values but rather increased their variances, i.e., wider parameter ranges were needed to accommodate the two different datasets.

Therefore, there is no question we are really learning and improving our solutions by assimilating the new dataset from the SCI protocol. Unfortunately, some parameters have not changed when moving from *P*_•_ to the *PO*_•_ populations. See, for instance, the cases of parameters *x*_2_ and *x*_3_. Together with the fact they have very large variances, these parameters are likely to be unidentifiable. At least, if we use only the I_CaL_ curve as data. However, it is worth noting from Figs 11 and 12 that the propagation of the uncertainties in the parameters [36] have a high impact on the waveforms of [*Ca*]_*i*_. Since both *x*_2_ and *x*_3_ are related to calcium-dependent inactivation, it is worth including both I_CaL_ and [*Ca*]_*i*_ in a future work that uses multi-ojective optimization tools, such as those described in [35, 37].

## Discussion

We have employed DE algorithms to replace the HH Gate-based description of the channels of two electrophysiology models of human cardiac cells Ten Tusscher and Panfilov [23], and Stewart et al. [24], by a new MC-based model. We selected the new parameters to mimic the dynamics of the original cardiac models, giving rise to two new models that fit the original AP and calcium concentrations, among other model characteristics. We show the importance of using the best fitting and an ensemble of a set of parameter values to reproduce a different scenario that considers the suppression of the calcium inactivation.

The presence of MC structures in the cardiac models brings several possibilities of studies capable of generating simulations over different scales, from subcellular up to tissue scale. This possibility allows computational models to help investigate pathologies that have their cause in the subcellular scale but can affect whole-cell and AP propagation. The use of MC-based formulations has been widespread to simulate Ryanodine Receptors dynamics of different cells and species. However, this approach was not too common for the I_CaL_ dynamics, particularly with respect to human cardiac models. In this direction, this study presented a new MC-based I_CaL_ model for two consolidated cardiac models for humans, Ten Tusscher and Panfilov [23], and Stewart et al. [24]. The computational approach employed to generate the MC-based models is based on an Evolutionary and Population-based algorithm. From the fitting results, we check that the newly proposed models, based on the fitting solutions, reproduce the Gate-based original outputs properly. This means that the new MC-based models maintained the original cell scale simulations. Furthermore, using the MC in the I_CaL_ formulations opens the possibility of simulating subcellular conditions and checking the response of the whole cell and, eventually, cardiac tissue. Using a multi-objective algorithm combined with Uncertainty Quantification analysis may improve our first findings and generate more reliable and consistent models.

Standard electrophysiological models, for example Ten Tusscher and Panfilov [23] and Stewart et al. [24], are usually fitted to a particular set of experiments. The resulting parameter values of such models may not be robust to changes due to a particular pathological condition. To overcome this issue, here we fit a population of Markov Chain-based models that conveniently fit the original experiments within a given tolerance that reflects both model discrepancy and biological variability. We have shown that the same population of solutions encountered by the DE was robust enough to reproduce a new set of experimental data associated with the SCI protocol. One may consider the SCI protocol discussed above as one limit condition and analyze intermediate situations as presented in [38].

Populations of models have been successfully used to represent inter-patient variability of cardiac electrophysiological data [3]. However, it is worth highlighting that the goals and tools used here are different ones. Our main goal is to acknowledge the existence of model discrepancy and identifiability issues, and use higher tolerances after the fitting process to avoid fitting parameters overly precisely, i.e., overfitting. Moreover, the tool used for this was a Differential Evolution algorithm based on evolutionary processes to select a population of parameters that satisfy our error tolerance. It is worth mentioning that considering a sample of best solutions instead of selecting only the best single one does not define a consolidated optimization technique to suppress the overfitting issue. In our study, we did not apply any consolidated technique during the DE process. Instead, we simply selected more solutions than the best one found by the algorithm; then we analyzed how these samples of solutions performed when we applied in a different condition. It is the simplest and computationally cheapest way to avoid one overfitted solution in the possible solutions. Probably, using a consolidated technique such as Early-stopping or Expansion of the set of data [39] in the evolutionary algorithm process will provide more reliable no-overfitted solutions.

One limitation of our method is related to the size of the population of solutions. The choice of the population size was arbitrary but driven by the acknowledgment of model discrepancy [34] and the seek of model generalization. Therefore, we include all solutions with errors below a certain level within the biological variability found in the experiments, 10 − 20%.

For the study presented here, the derived calibrated population was able to replicate new experimental data. The population was calibrated with control data and reproduced different data from pathological conditions (SCI protocol). Nevertheless, the population’s average error of some biomarkers (see Table 1) increased from 4–5%, for the control case (or training data), to 20–30%, for the new data (test data). In the case this new level of error is unacceptable, the solution would be to repeat the calibration process including the new data via multi-objective optimization tools, such as those described in [35, 37], and further speedup up the process with emulators as described in [40].

Our method allows us to relate any animal model with a Markov chain of the LCC that can then be fitted to new experimental data with the limitation that possible structural intrinsic differences between the HH Gate-based, used to generate the training and test data, and the MC-based description of the channels can be present. Nevertheless, this difference generates a model discrepancy that is very likely to happen in real-life experimental data. Therefore, we believe this structural difference between the models has positively contributed to the study and further supports our conclusions. It is also important to clarify that the use of our method to link experimental and whole-cell models is restricted to those models where the I_CaL_ formulation can be arranged as *I*_*CaL*_ = *I*_*max*_ × *O*, where *I*_*max*_ is the maximum current conductance; and *O* represents the portion of the opening fraction of the ionic channels. Besides the TP and ST models, the method can be applied in the models described in [41, 42]. On the other hand, we can not apply the proposed methods in the models presented in [38, 43].

Besides the possibility of directly relating new experimental calcium whole-cell data with a Markov model of the LCC, our method also opens a new line of research where direct links between whole-cell model data and subcellular mechanism can be investigated. It is important to remember that calcium is driven by subcellular interactions at the micron level where LCC and RyR form couplons with different cluster size and important heterogeneity [44]. This means that each couplon will have different probabilistic openings. The relation between the local parameters of the Markov-chain that could reproduce the couplon behavior and the parameters of the single Markov-model found with whole-cell data can now be addressed. Analyzing how a single set of parameters in the Markov-model affects the probabilities of openings depending on cluster size should be the focus of future investigations to entangle how the local release probabilities affect the global calcium cycling of the cell.

In summary, we developed novel Markov Chain models for the L-type Calcium current that reproduced the electrophysiology of two human models for the ventricular and Purkinje cells. In addition, instead of presenting a single model, we presented a population of models based on different solutions found by a robust fitting process. These models could properly reproduce very different protocol data. In particular, we show the importance of using a population of models to obtain proper results when considering a second protocol that mimics the condition of Calmodulin mutations associated with long QT syndrome. The use of populations of solutions was crucial for addressing model discrepancy, identifiability issues, and avoiding fitting parameters overly precisely, i.e., overfitting.

## Supporting information

S1 FigThreshold used for the selection of the individuals.Representation of the threshold (red dashed line) considered to select the individuals to be part of the population *P*_•_ for the respective (A) TP Model, and (B) ST Model. For both models, we selected the best 300 from the 5000 possibilities (or 6% of all the individuals). The worst individual selected to compose the population *P*_*TP*_, $x_{TP}^{w}$, obtained $F(x_{TP}^{w})=15.1\%$. The worst individual selected to compose the population *P*_*ST*_, $x_{ST}^{w}$, obtained $F(x_{ST}^{w})=16\%$. The light red area represents the solutions in the rather linear relation. The light blue area represents the solutions in the rather exponential relation.(TIF)

S1 Appendix New Markov chain-based rates. This appendix introduces the new I_CaL_ MC-based equations and rates.
(PDF)

S2 Appendix Frequency rate robustness. This appendix presents the outputs of the MC-based versions of the TP and ST models simulated under different pacing rates.
(PDF)

S3 Appendix Model robustness. This appendix presents an analysis of robustness the MC-based versions of the TP and ST models.
(PDF)

S1 Data (ZIP)
