# Supplementary figures and images for: An ensemble of parameters from a robust Markov-based model reproduces L-type calcium currents from different human cardiac myocytes
Source: PLoS One. 2022 Apr 5;17(4):e0266233. doi: 10.1371/journal.pone.0266233 (PMC8982880; doi:10.1371/journal.pone.0266233)

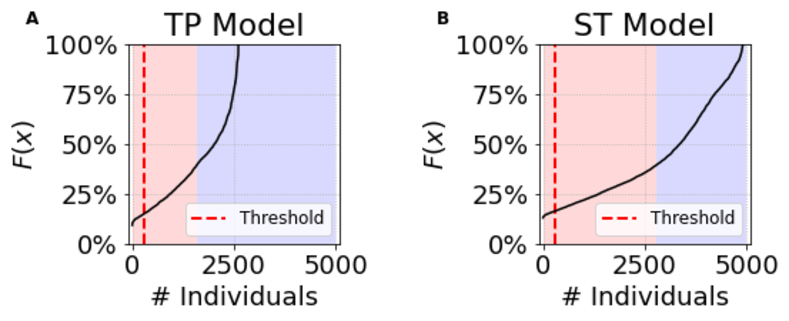

Supplement: S1 Fig — Representation of the threshold (red dashed line) considered to select the individuals to be part of the population P• for the respective (A) TP Model, and (B) ST Model. For both models, we selected the best 300 from the 5000 possibilities (or 6% of all the individuals). The worst individual selected to compose the population PTP, xTPw, obtained F(xTPw)=15.1%. The worst individual selected to compose the population PST, xSTw, obtained F(xSTw)=16%. The light red area represents the solutions in the rather linear relation. The light blue area represents the solutions in the rather exponential relation. (TIF) [file pone.0266233.s001.tif]
