# Supplementary material for: An ensemble of parameters from a robust Markov-based model reproduces L-type calcium currents from different human cardiac myocytes
Source: PLoS One. 2022 Apr 5;17(4):e0266233. doi: 10.1371/journal.pone.0266233 (PMC8982880; doi:10.1371/journal.pone.0266233)
Supplement: S1 Appendix — This appendix introduces the new ICaL MC-based equations and rates. (PDF) [file pone.0266233.s002.pdf]

S1 Appendix. New Markov chain-based rates. This appendix introduces the new $\mathrm{I_{CaL}}$ MC-based equations and rates. We used the same MC topology used to simulate the $\mathrm{I_{CaL}}$ phenomenon in [1]. They use seven states disposed of three layers being the above one layer, the Calcium Dependence layer. The other two layers are only Voltage Dependence. Fig 1 presents the new MC illustration.

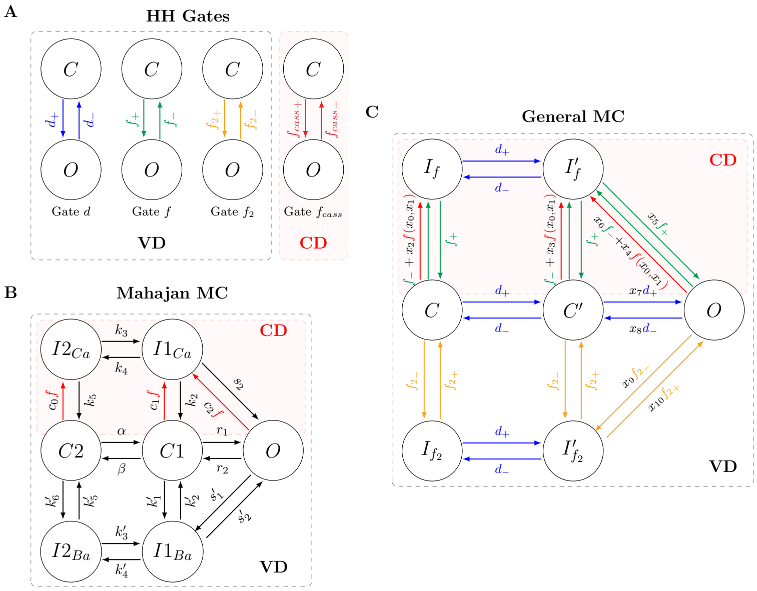

Fig 1. Schematic representations of the three markov chain structures considered in this study. A: The four independent Markov Chains for each gate of the models Ten Tusscher and Panfilov [2] or Stewart et al. [3] considering only two possible states, Open (O) and Close (C) for each one. B: The original structure of the Markov Chain used by Mahajan et al. [1] to simulate the $\mathrm{I_{CaL}}$ phenomenon. C: The proposed Markov Chain as a combination of the Hodgkin-Huxley formalism rates and the Mahajan et al. [1] topology, to replace the gates in the Ten Tusscher and Panfilov [2] and Stewart et al. [3] models. The MC transitions generated considering the HH gates d, f, and f_2 are shown respectively in blue, green, and yellow. The calcium-dependent function f and the rates associated with the calcium concentration are shown in red. The set of parameters $\boldsymbol{x}$ used to fit both calcium-dependent rates (parameters x_0 to x_4) and voltage-dependence rates (parameters x_5 to x_{10}) are shown in black.

The rates $\bullet_+$, and $\bullet_-$, that compose the MC rates, are calculated based in the Hodgkin-Huxley formalism, and they were obtained from the respective original models, Ten Tusscher and Panfilov [2], and Stewart et al. [3]. Table in 1 presents the equations considered in the rates calculations. As the Stewart et al. [3] study was generated based in the Ten Tusscher and Panfilov [2], it is important to highlight that both models share almost all equations that compose their $\mathrm{I_{CaL}}$ formulations. The difference between them are represented by the Table in 1 as the parameters α, β, γ, and δ.

Considering the Calcium-Dependence rate, represented by $f = f(x_0, x_1, c)$, it was adapted from [1]. The original $f(c)$ function used in [1] reads

$$f(c) = \frac{1}{1 + (\bar{c_p}/c)^3}, \tag{1}$$

where c is the subcellular calcium concentration ($[Ca]_{ss}$), and $\bar{c_p}$ is a threshold for calcium dependence.

Table 1. Formulations of the generalized markov-chain rates.

TP and ST Models				
Rates	$\bullet_{inf}$	$\tau_\bullet$	$\bullet_+$	$\bullet_-$
d	$d_{inf} = \frac{1}{1+e^{(-8-V)/7.5}}$	$\tau_d = A_d \times B_d + C_d$	$d_+ = d_{inf}/\tau_d$	$d_- = (1 - d_{inf})/\tau_d$
f	$f_{inf} = \frac{1}{1+e^{(V+20)/7}}$	$\tau_f = A_f + B_f + C_f$	$f_+ = f_{inf}/\tau_f$	$f_- = (1 - f_{inf})/\tau_f$
f_2	$f_{2inf} = \frac{0.67}{1+e^{(V+35)/7}} + 0.33$	$\tau_{f_2} = A_{f2} + B_{f2} + C_{f2}$	$f_{2+} = f_{2inf}/\tau_{f_2}$	$f_{2-} = (1 - f_{2inf})/\tau_{f_2}$
Rates		$A_\bullet$	$B_\bullet$	$C_\bullet$
d		$A_d = \frac{1.4}{1+e^{(-35-V)/13}} + 0.25$	$B_d = \frac{1.4}{1+e^{(V+5)/5}}$	$C_d = \frac{1}{1+e^{(50-V)/20}}$
f		$A_f = 1102.5e^{(-(V+27)^2/225)}$	$B_f = \frac{200}{1+e^{(13-V)/10}}$	$C_f = \frac{180}{1+e^{(V+30)/10}} + 20$
f_2		$A_{f_2} = \alpha e^{-(V+\beta)^2/\gamma}$	$B_{f_2} = \frac{31}{1+e^{(25-V)/10}}$	$C_{f_2} = \frac{\delta}{1+e^{(V+30)/10}}$

Equations used to calculate the rates $\bullet_+$, and $\bullet_-$ that compose the generalized MC used in the MC-based version of the models [2] (TP Model), and [3] (ST Model). For TP Model: $\alpha = 600$, $\beta = 25$, $\gamma = 170$, and $\delta = 16$. For ST Model: $\alpha = 562$, $\beta = 27$, $\gamma = 240$, and $\delta = 80$.

Fitting parameters. The new Markov Chain was adapted from the HH formalism presented in the original Ten Tusscher and Panfilov [2], and Stewart et al. [3] models combined with the previous I_{CaL} formulation originally proposed in [1]. As an adaptation, a fitting process was necessary to adequate the novel MC-based I_{CaL} dynamics to the original values.

To introduce the adjustable capacity into the new MC, we selected the main rates associated with the channel opening dynamics. For the portion of the dynamics associated with the calcium-dependence, we introduced the adjustable feature in two ways. The first one was to add two fitting parameters, x_0, and x_1, in the original Mahajan et al. [1] f function, Eq (1), as the simplest linear combination with the original values. Thereby, our adjustable calcium-dependence rate reads

$$f(c, x_0, x_1) = \frac{1}{1 + (x_0 \bar{c_p}/c)^{x_1 3}}. \quad (2)$$

Furthermore, to open more possibilities for the fitting algorithm to adjust the calcium-dependence, we also added, as the simplest linear combination, one parameter multiplying the f function in each transition where it is inserted. As we have three transitions which are composed by the calcium-dependence f function, $C \to I_f$, $C' \to I'_f$, and $O \to I'_f$, we added three new parameters, x_2, x_3 , and x_4. Fig 1 presents the calcium-dependence rates in red color.

For the portion of the dynamics associated with the voltage-dependence, we selected the main transitions that are directly associated with the MC opening state, $I'_f \rightleftarrows O$, $C' \rightleftarrows O$, and $I'_{f2} \rightleftarrows O$. For each rate which composes the transitions, we introduced a fitting parameter x as the simplest linear combination. Thus, the fitting process is capable to act directly in the main variable of interest: the MC Opening fraction. This method generated a total of seven parameters (x_5 up to x_{10}).

Therefore, to introduce the fitting possibility in the new MC, we inserted five parameters associated with the calcium-dependence, and other six parameters associated with the voltage-dependence dynamics.

References

1. Mahajan A, Shiferaw Y, Sato D, Baher A, Olcese R, Xie LH, et al. A rabbit ventricular action potential model replicating cardiac dynamics at rapid heart rates. Biophysical journal. 2008;94(2):392–410.

2. Ten Tusscher KH, Panfilov AV. Alternans and spiral breakup in a human ventricular tissue model. American Journal of Physiology-Heart and Circulatory Physiology. 2006;291(3):H1088–H1100.

3. Stewart P, Aslanidi OV, Noble D, Noble PJ, Boyett MR, Zhang H. Mathematical models of the electrical action potential of Purkinje fibre cells. Philosophical Transactions of the Royal Society A: Mathematical, Physical and Engineering Sciences. 2009;367(1896):2225–2255.
