# Supplementary material for: An ensemble of parameters from a robust Markov-based model reproduces L-type calcium currents from different human cardiac myocytes
Source: PLoS One. 2022 Apr 5;17(4):e0266233. doi: 10.1371/journal.pone.0266233 (PMC8982880; doi:10.1371/journal.pone.0266233)
Supplement: S2 Appendix — This appendix presents the outputs of the MC-based versions of the TP and ST models simulated under different pacing rates. (PDF) [file pone.0266233.s003.pdf]

S2 Appendix. Frequency rate robustness. One important biological phenomenon associated with the cardiac cells is the rate of the stimulus to which the cells are submitted. Normally, the mathematical models consider the frequency of 1Hz, but the response of the models under different stimulus rates is crucial to maintain the robustness in representing the cardiac cells in different biological conditions.

Therefore, we tested the best solution of the respective populations $P_\bullet$, $\boldsymbol{x^b_{TP}}$, and $\boldsymbol{x^b_{ST}}$, fitted for each respective target models, Ten Tusscher and Panfilov [1], and Stewart et al. [2], to check if the solutions maintained the rate-dependence responses presented in the original models. Fig in 1 shows the restitution curves obtained by the best solutions $\boldsymbol{x^b_\bullet}$ compared with the curves obtained simulating the respective target models, Ten Tusscher and Panfilov [1], and Stewart et al. [2]. To generate the restitution curves, we adopted a Sweep protocol. Beginning from a stimulus period of 1000 ms, after every 100 consecutive pulses, we decrease this period by 50 ms. Therefore, we simulated 100 pulses using a stimulus period of 1000 ms, the next 100 pulses were generated using a stimulus period of 950ms; and we decreased this value until 300 ms. To compute the APD_{90} at each respective period value $p \in P = \{300, 350, ..., 950, 1000\}$, we considered the 100th AP curve.

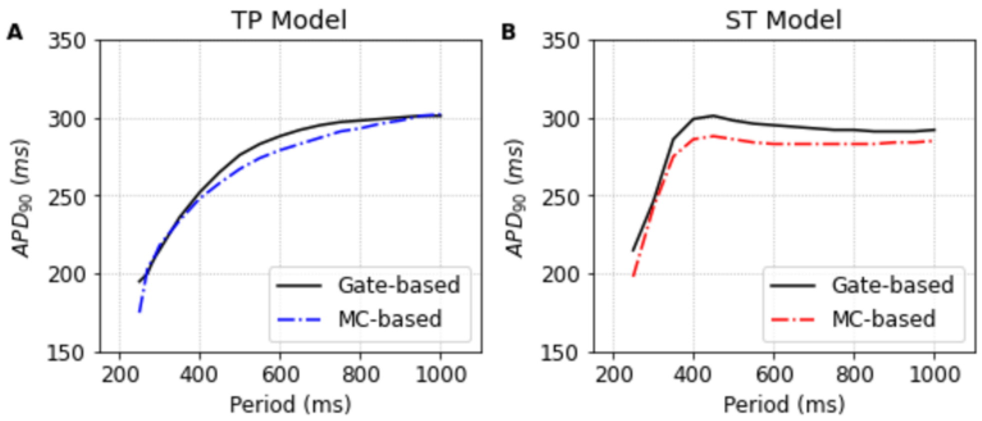

Fig 1. Restitution curves. Comparison between the restitution curves obtained by simulating the best solution of the respective population of solutions P_{TP}, $\boldsymbol{x^b_{TP}}$, and P_{ST}, $\boldsymbol{x^b_{ST}}$, compared with the same curves obtained simulating the respective original models TP, and ST unde Full protocol. A: TP Model. B: ST Model.

As can be seen in Fig 1, the solutions found by the DE obtained satisfactory results when simulated under faster pacing rates. So, as we could see, the MC-based version of both Ten Tusscher and Panfilov [1], and Stewart et al. [2] models were able to maintain the frequency rate robustness presented in the respective original models.

References

1. Ten Tusscher KH, Panfilov AV. Alternans and spiral breakup in a human ventricular tissue model. American Journal of Physiology-Heart and Circulatory Physiology. 2006;291(3):H1088–H1100.

2. Stewart P, Aslanidi OV, Noble D, Noble PJ, Boyett MR, Zhang H. Mathematical models of the electrical action potential of Purkinje fibre cells. Philosophical Transactions of the Royal Society A: Mathematical, Physical and Engineering Sciences. 2009;367(1896):2225–2255.
