# Supplementary material for: An ensemble of parameters from a robust Markov-based model reproduces L-type calcium currents from different human cardiac myocytes
Source: PLoS One. 2022 Apr 5;17(4):e0266233. doi: 10.1371/journal.pone.0266233 (PMC8982880; doi:10.1371/journal.pone.0266233)
Supplement: S3 Appendix — This appendix presents an analysis of robustness the MC-based versions of the TP and ST models. (PDF) [file pone.0266233.s004.pdf]

S3 Appendix. Model robustness. A computational model usually has the main objective to answer one or a couple of questions. It is completely acceptable since a model, by the premise, is a representation or a simplification of an object or phenomenon. All the other behaviors or answers that can not be analyzed by a model compose its limitations. Thus, we can associate the robustness of a model by considering the capacity of this model to reproduce more or fewer behaviors or conditions related to its nature. As part of this context, the cardiac models present their intrinsic limitations.

In our study we adapted two consolidated cardiac models, TP and ST, replacing its Gate-based I_{CaL} formulation with a Markov Chain-based. Once we are altering the original formulations, it is important to check if the robustness present in the original models were preserved. In the main manuscript text, we analyzed the fitting procedure robustness. We showed that a single fitting could find parameters sets capable to reproduce both Full (healthy cell condition) and Suppressed Calcium Inactivation (pathological cell condition) protocols.

In this Appendix, we will analyze the robustness of the MC-based models by doing a second fitting procedure to reproduce a different and specific protocol. For that, we select an S1S2 protocol composed of two pacing rates while its simulation. The protocol consists to stimulate the model using a pacing rate of 1Hz during the first 10s; after that, we change this rate to 2Hz and still stimulate the model by 1s. At the end of the total of 11s of simulation, we have a total of 12 pulses. In Fig. 1 we show an illustration of the I_{Stim} and the I_{CaL} currents obtained under the S1S2 protocol.

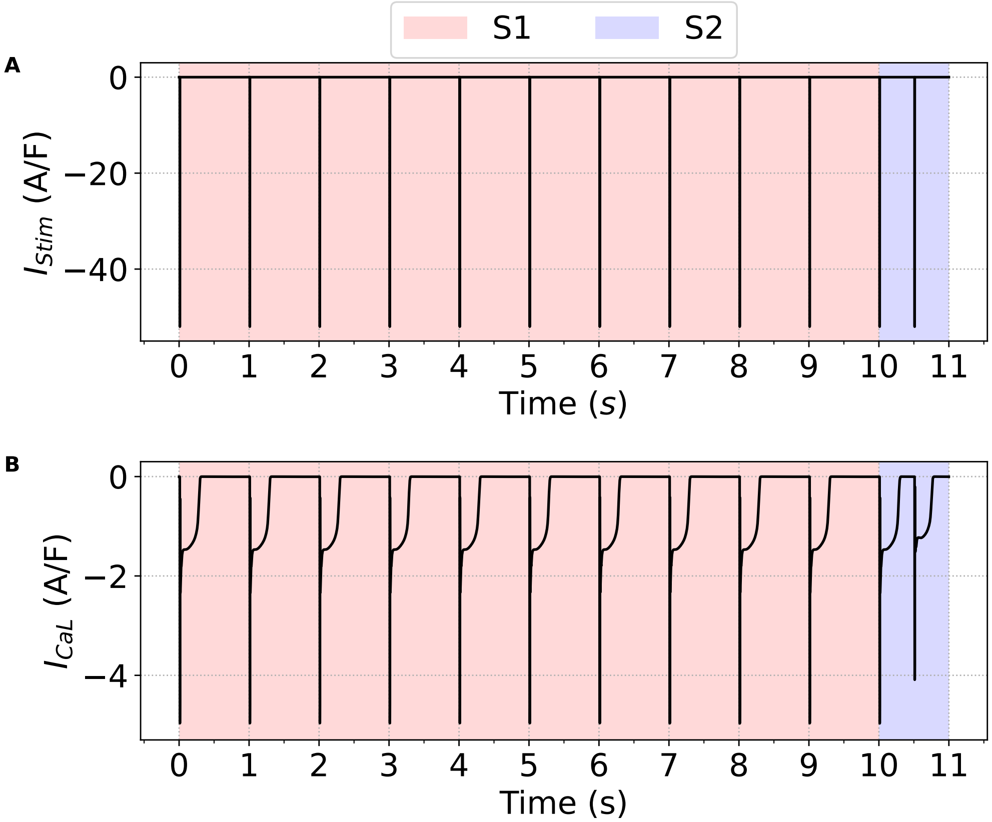

Fig 1. S1S2 Protocol. Illustration of the S1S2 protocol applied in the I_{Stim} (A) and the I_{CaL} (B) currents. For the S1S2 protocol, we stimulated the cell at 1Hz while the first 10s (red area) of simulation and then, by 1s, we changed the frequency to 2Hz (blue area).

Differently, as we did in the main manuscript text, where we selected the 11th pulse, now, to get the effect of the S2 portion of the protocol, we will consider as our target the 12th pulse. So, we defined our fitness function F(x) as

$$NF(\boldsymbol{x}) = \sqrt{\sum_{t=10.5s}^{11s} \frac{(I_{CaL_{MC}}(\boldsymbol{x}, t) - \overline{I_{CaL_{HH}}}(t))^2}{\overline{I_{CaL_{HH}}}(t)^2}}, \tag{1}$$

where $\overline{I_{CaL_{HH}}}$ is the calcium current of the HH gate-based model and the $I_{CaL_{MC}}$ is the calcium current generated by the new MC-based model using the parameter set $\boldsymbol{x}$. Please, note that, for this protocol fitting, we considered the $\mathrm{I_{CaL}}$ curve between the times 10.5s and 11s.

As can be seen in Fig. 2 and in Fig. 3, the new fitting procedures were able to find a solution for reproducing each respective models, TP and ST, simulated under the S1S2 protocol. For the TP model under the S1S2 protocol, the best solution found in the new fitting procedure, $\boldsymbol{x^{sb}_{TP}}$, obtained a fitness error $NF(\boldsymbol{x^{sb}_{TP}})$ of 7.5%. Just to compare, the solutions $\boldsymbol{x^{b}_{TP}}$ and $\boldsymbol{x^{o}_{TP}}$, presented in the main text of the manuscript, obtained, respectively, 10.7% and 15.2% of the fitness error, $NF(\boldsymbol{x^{b}_{TP}})$ and $NF(\boldsymbol{x^{o}_{TP}})$, when simulated under the same S1S2 protocol. For the ST model under the S1S2 protocol, the best solution found in the new fitting procedure, $\boldsymbol{x^{sb}_{ST}}$, obtained a fitness error $NF(\boldsymbol{x^{sb}_{ST}})$ of 8.7%. In comparison, the solutions $\boldsymbol{x^{b}_{ST}}$ and $\boldsymbol{x^{o}_{ST}}$, presented in the main text of the manuscript, obtained, respectively, 9.2% and 20.3% of the fitness error, $NF(\boldsymbol{x^{b}_{ST}})$ and $NF(\boldsymbol{x^{o}_{ST}})$, when simulated under the same S1S2 protocol.

Therefore, considering the second S1S2 fitting results, we can conclude that the method used in this paper were able to fit and reproduce a different protocol experiment as expected by the original models. Thus, the proposed method was capable to maintain the robustness of the original TP and ST models in their MC-based versions.

References

1. Ten Tusscher KH, Panfilov AV. Alternans and spiral breakup in a human ventricular tissue model. American Journal of Physiology-Heart and Circulatory Physiology. 2006;291(3):H1088–H1100.

2. Stewart P, Aslanidi OV, Noble D, Noble PJ, Boyett MR, Zhang H. Mathematical models of the electrical action potential of Purkinje fibre cells. Philosophical Transactions of the Royal Society A: Mathematical, Physical and Engineering Sciences. 2009;367(1896):2225–2255.

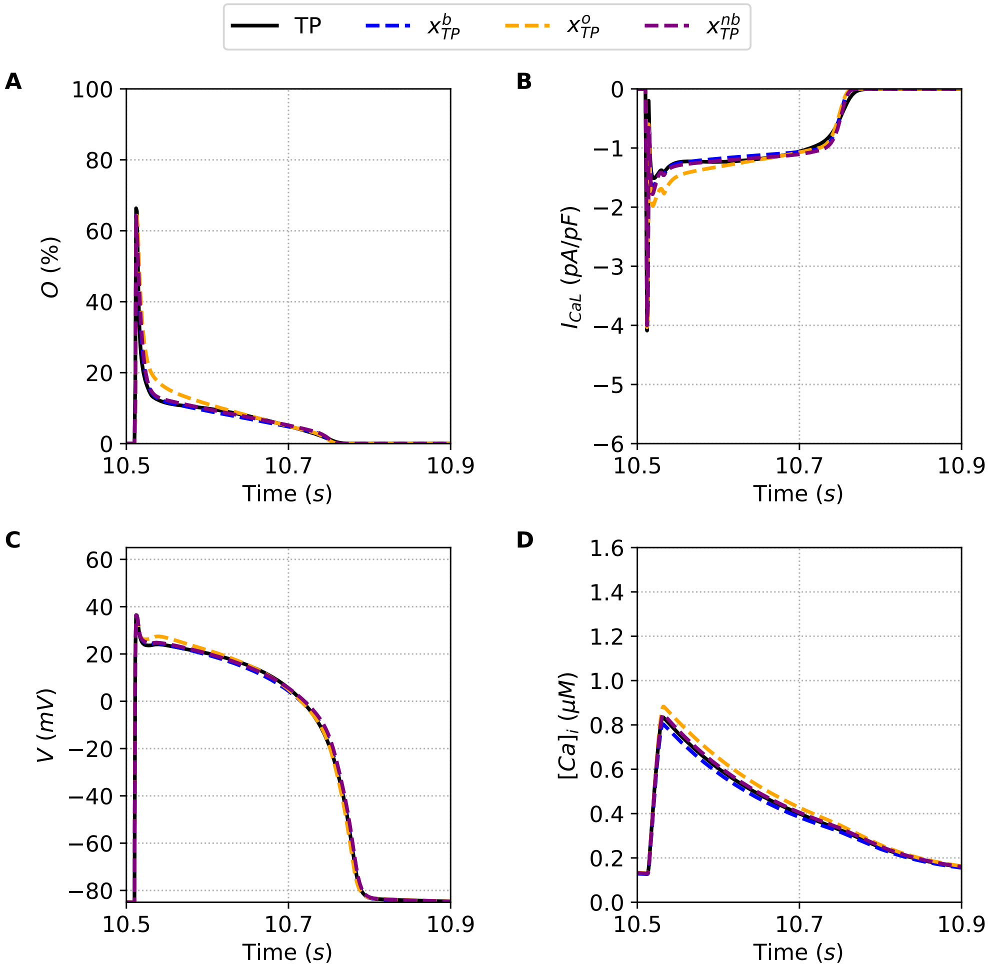

Fig 2. Outputs of the S1S2 protocol for the Ten Tusscher and Panfilov [1] model. Traces obtained using the best solution $\boldsymbol{x_{TP}^{nb}}$ (dashed purple line), alongside the best solution of the population P_{TP}, $\boldsymbol{x_{TP}^{b}}$ (dashed blue line), and the best overall solution $\boldsymbol{x_{TP}^{o}}$ (dashed orange line) in comparison with the original Ten Tusscher and Panfilov [1] model (black line) under the S1S2 protocol.

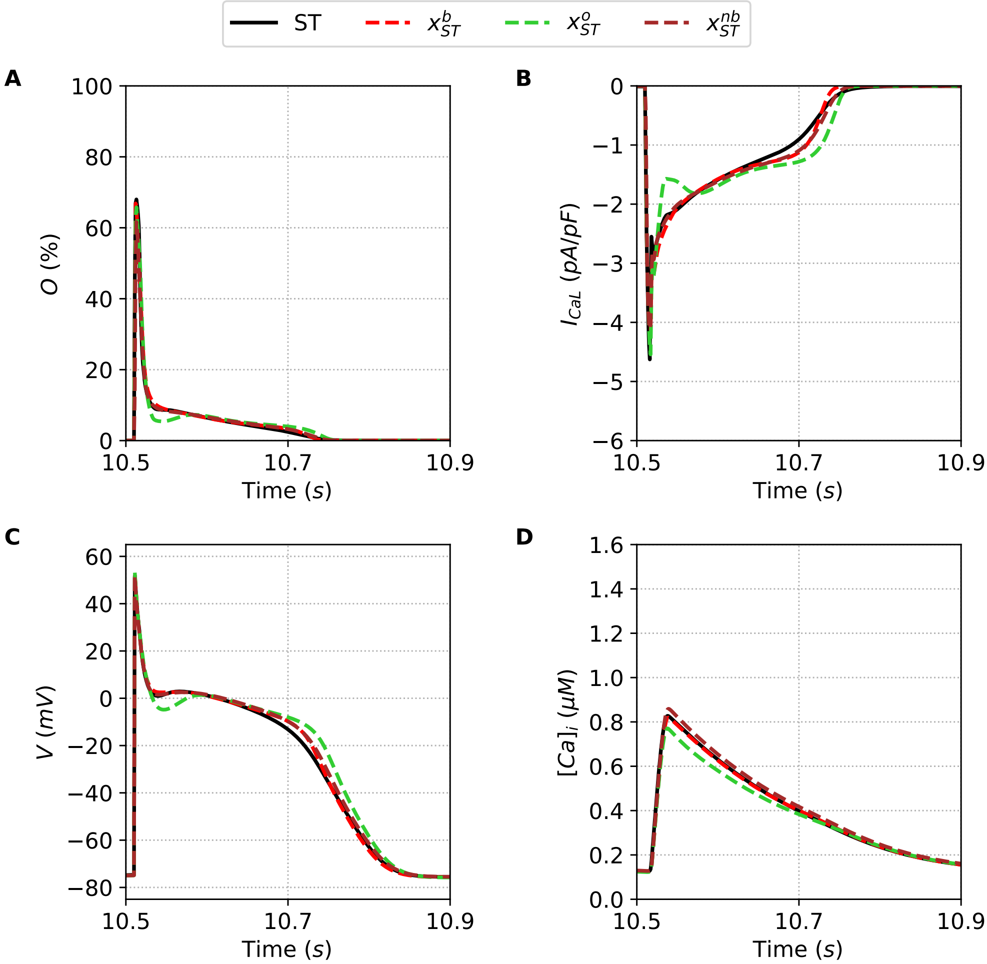

Fig 3. Outputs of the S1S2 protocol for the Stewart et al. [2] model. Traces obtained using the best solution $\boldsymbol{x^{nb}_{ST}}$ (dashed brown line), alongside the best solution of the population P_{ST}, $\boldsymbol{x^{b}_{ST}}$ (dashed red line), and the best overall solution $\boldsymbol{x^{o}_{ST}}$ (dashed green line) in comparison with the original Stewart et al. [2] model (black line) under the S1S2 protocol.
